# *Gekko japonicus* genome reveals evolution of adhesive toe pads and tail regeneration

**DOI:** 10.1038/ncomms10033

**Published:** 2015-11-24

**Authors:** Yan Liu, Qian Zhou, Yongjun Wang, Longhai Luo, Jian Yang, Linfeng Yang, Mei Liu, Yingrui Li, Tianmei Qian, Yuan Zheng, Meiyuan Li, Jiang Li, Yun Gu, Zujing Han, Man Xu, Yingjie Wang, Changlai Zhu, Bin Yu, Yumin Yang, Fei Ding, Jianping Jiang, Huanming Yang, Xiaosong Gu

**Affiliations:** 1 JS Key Laboratory of Neuroregeneration, Co-innovation Center of Neuroregeneration, Nantong University, Nantong 226001, China; 2 Beijing Genomics Institute, BGI-Shenzhen, Shenzhen 518083, China; 3 Chengdu institute of biology, Chinese Academy of Sciences, Chengdu 610041, China; 4 James D. Watson Institute of Genome Sciences, Hangzhou 610041, China

## Abstract

Reptiles are the most morphologically and physiologically diverse tetrapods, and have undergone 300 million years of adaptive evolution. Within the reptilian tetrapods, geckos possess several interesting features, including the ability to regenerate autotomized tails and to climb on smooth surfaces. Here we sequence the genome of *Gekko japonicus* (Schlegel's Japanese Gecko) and investigate genetic elements related to its physiology. We obtain a draft *G. japonicus* genome sequence of 2.55 Gb and annotated 22,487 genes. Comparative genomic analysis reveals specific gene family expansions or reductions that are associated with the formation of adhesive setae, nocturnal vision and tail regeneration, as well as the diversification of olfactory sensation. The obtained genomic data provide robust genetic evidence of adaptive evolution in reptiles.

## Abstract (web summary)

Geckos are small, agile reptiles with nocturnal habits. Here, the authors sequence the genome of the Schlegel's Japanese Gecko and reveal gene family expansions and reductions associated with formation of adhesive setae, nocturnal vision, tail regeneration, and diversification of olfactory sensation.

## Graphical abstract

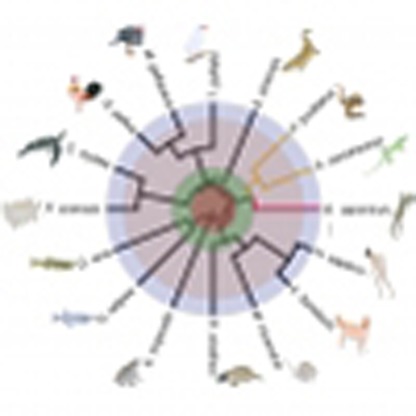

Reptiles diverged from early tetrapods in the late *Carboniferous* period approximately 310–320 million years ago (Myr ago)^1^. Since then, these ancient amniotes have spread across the world and adopted diverse morphologies and habitats (aquatic and terrestrial)^2^. More than 10,000 reptile species have been documented and they are classified into the following four orders: Crocodilia, Testudines, Squamata and Sphenodontia^3^. Squamate reptiles represent the most diverse radiation of terrestrial vertebrates and are traditionally split into two major clades, Iguania and Scleroglossa^3^,^4^. The latter clade includes the terrestrial Gekkonidae, which consists of ∼1,450 species in 118 genera and comprises 25% of all described lizard species^5^. Geckos have evolved in a terrestrial niche, where selective pressure^6^ has resulted in traits such as small body size, agility and nocturnal habits. Most gecko species possess adhesive toe pads, which enable them to capture live food more easily, and flee from their predators by scaling vertical or even inverted surfaces^7^. This ability is due to the presence of setae, microscopic hair-like outgrowths of the superficial layer of the subdigital epidermis, which comprise the primary components of the adhesive apparatus^7^,^8^. The most interesting and physiologically significant trait in geckos is their ability to voluntarily shed or autotomize their tails to escape from attack, they then regenerate a new tail^9^. Given these interesting characteristics, geckos have been used in studies on regenerative processes, and their adhesive mechanism has been examined for the development of bio-inspired technologies^10^. The availability of genome sequence data would significantly contribute to deciphering the evolutionary events related to lineage-specific anatomical adaptations.

To date, the genomes of several reptilian species, including species from Squamata reptiles (*Anolis carolinensis*, *Python molurus bivittatus* and *Ophiophagus hannah*), Crocodylia reptiles (*Alligator sinensis*, *Alligator mississippiensis*, *Gavialis gangeticus* and *Crocodylus porosus*) and Testudines reptiles (*Chelonia mydas*, *Pelodiscus sinensis* and *Chrysemys picta bellii*) have been successfully sequenced^11^,^12^,^13^,^14^,^15^. These results have filled the genomic gap between amphibians and birds by providing genomic information on a wide variety of morphologically and physiologically distinct species. However, genomic data from the species comprising the family Gekkonidae, which is an important clade of Lepidosauria and one of the earliest branches off the squamata phylogenetic tree, have not yet been acquired. In this study, the genome of *G. japonicus* (Schlegel's Japanese Gecko) is sequenced and annotated, which provides valuable insights into the adaptive evolution of geckos as well as the genomic basis of their characteristic traits. For example, our data reveals that the expansion of *β-keratin* gene family is essential to the clinging ability of *G. japonicus*, and the evolution of the *opsin* gene is correlated with its visual adaptation. Moreover, some positive selected genes (PSGs) potentially involved in the tail regeneration are identified as well. In addition, developing a genomic resource associated with geckos is helpful in understanding the evolutionary history of Lepidosauria.

## Results

### Sequencing and annotation of the *G. japonicus* genome

The genome of an adult male *G. japonicus* was sequenced and assembled (Supplementary Figs 1–3 and Tables 1–3). The draft genome sequence of *G. japonicus* was 2.55 Gb in size, ∼50% larger than that of *Anolis carolinensis*, a lizard that belongs to the Iguania of Squamata, making it the largest sequenced genome to date among all reptiles with available genome data^11^,^12^,^13^,^14^. The assembly quality was assessed using 10 fosmid clones and RNA-Seq data (Supplementary Fig. 4 and Tables 4–6).

Repeated elements were annotated using Repeat Masker program^16^. The repeats comprised 48.94% of the genome, and most were transposable elements, making up 48.02% of the assembly (Supplementary Fig. 5 and Tables 7 and 8). The GC content in *G. japonicus* genome was about 45.5%, which is slightly higher than in genome of other amniotes (for example, *Anolis. carolinensis*, 40.3%; *Gallus gallus*, 41.5%; *Homo sapiens*, 40.8%). The GC content was primarily distributed in introns, the intergenic regions and CDS regions (Supplementary Figs 6 and 7). Collectively, the above data indicate that the large genome size of *G. japonicus* may primarily result from the greater abundance of repeated sequences compared with other genomes, such as that of *A. carolinensis* (Supplementary Tables 9–12).

A total of 22,487 coding regions and 1,302 non-coding RNAs were predicted in the *G. japonicus* genome (Supplementary Tables 13–15), and ∼95.08% of the coding regions were functionally annotated (Supplementary Table 16). Then, the orthologous and paralogous genes were clustered and compared among different species background (Supplementary Figs 8–10 and Tables 17 and 18). The data revealed that *G. japonicus* had 11,513 orthologous gene pairs compared with *A. carolinensis*, and the mean identity reached up to 72.37% (Supplementary Fig. 9 and Table 18). A comparison of gene data among four reptiles (*G. japonicus*, *An. carolinensis*, *Al. sinensis* and *C. mydas*) revealed ∼13,478 orthologous gene families in total, of which 7,546 were shared by four species, 1,240 were specific to *G. japonicus*, 798 were specific to *An. carolinensis*, 911 were specific to *Al. sinensis* and 673 were specific to *C. mydas* (Supplementary Fig. 10). These species-specific unique orthologous may be involved in lineage-specific adaptations.

### Evolutionary analysis of the *G. japonicus* genome

**Figure 1:**
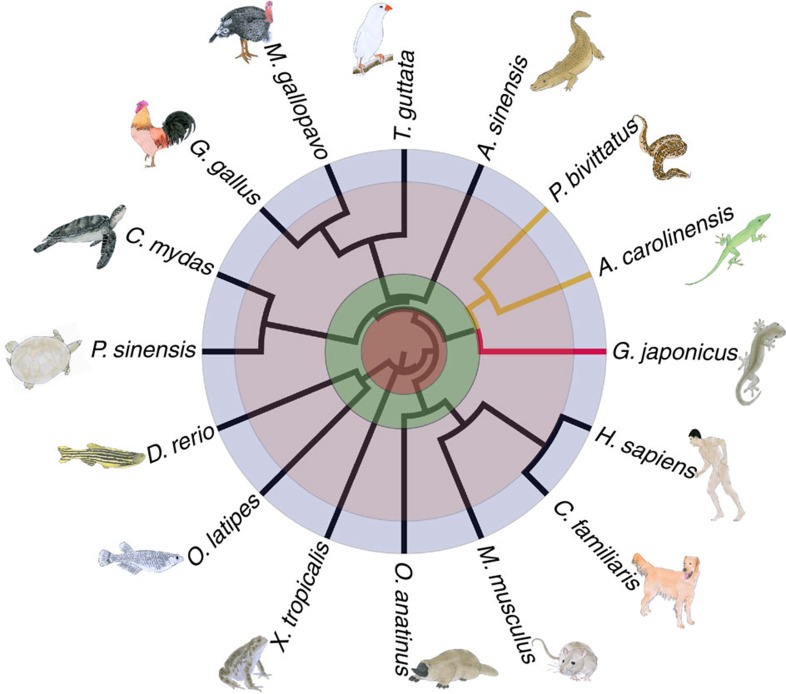
Phylogenetic analysis of the whole-genomes of 6 reptilian species and 10 additional vertebrate species. The species in the phylogenetic tree include *Danio rerio* (*D. rerio*), *Xenopus tropicalis* (*X. tropicalis*), *Chelonia mydas* (*C. mydas*), *Pelodiscus sinensis* (*P. sinensis*), *Alligator sinensis* (*A. sinensis*), *Python molurus bivittatus* (*P. bivittatus*), *Anolis carolinensis* (*A. carolinensis*), *Gekko japonicus* (*G. japonicus*), *Taeniopygia guttata* (*T. guttata*), *Gallus gallus* (*G. gallus*), *Ornithorhynchus anatinus* (*O. anatinu*s), *Canis familiaris* (*C. familiaris*), *Mus musculus* (*M. musculus*), *Homo sapiens* (*H. sapiens*), *Oryzias latipes* (*O. latipes*) and *Meleagris gallopavo* (*M. gallopavo*). Before the Permian period is represented in brown. The Permian period to the Triassic period is represented in green. The Triassic period to the Paleogene period is represented in purple. The Paleogene period to the present is represented in blue.

We assessed evolutionary relationships among morphologically and ecologically diverse reptiles by constructing a phylogenetic tree using the whole-genomes of 6 reptilian species and 10 other vertebrates. The results support the view that the species of Gekkota diverged early from the group containing Anolis and Python ∼200 Myr ago^17^, when Gondwanaland separated from Laurasia^18^. This time period is earlier than previous reported^19^, but later than the divergence of Sphenodon^20^. *A. carolinensis* clusters with *P. bivittatus* rather than with *G. japonicus*, indicating that *A. carolinensis* and *P. bivittatus* have a much closer genetic relationship, even though *P. bivittatus* and *G. japonicus* have traditionally been classified as scleroglossans^4^. The phylogenetic tree shows that the crocodilian lineage diverged from chelonian about 250 Myr ago and clusters in the same clade with birds (Fig. 1). A second phylogenetic tree constructed with conserved housekeeping proteins shows consistent results with these findings (Supplementary Fig. 11). The cladogram of whole-genomes clearly shows the evolutionary relationships among these selected amniotes, and the whole genome resource will reduce the ambiguities of phylogeny data when assessed by previous methods based on morphological characteristics or few genes^17^,^19^.

### Adaptive evolution of setae β-keratins in *G. japonicus*

**Figure 2:**
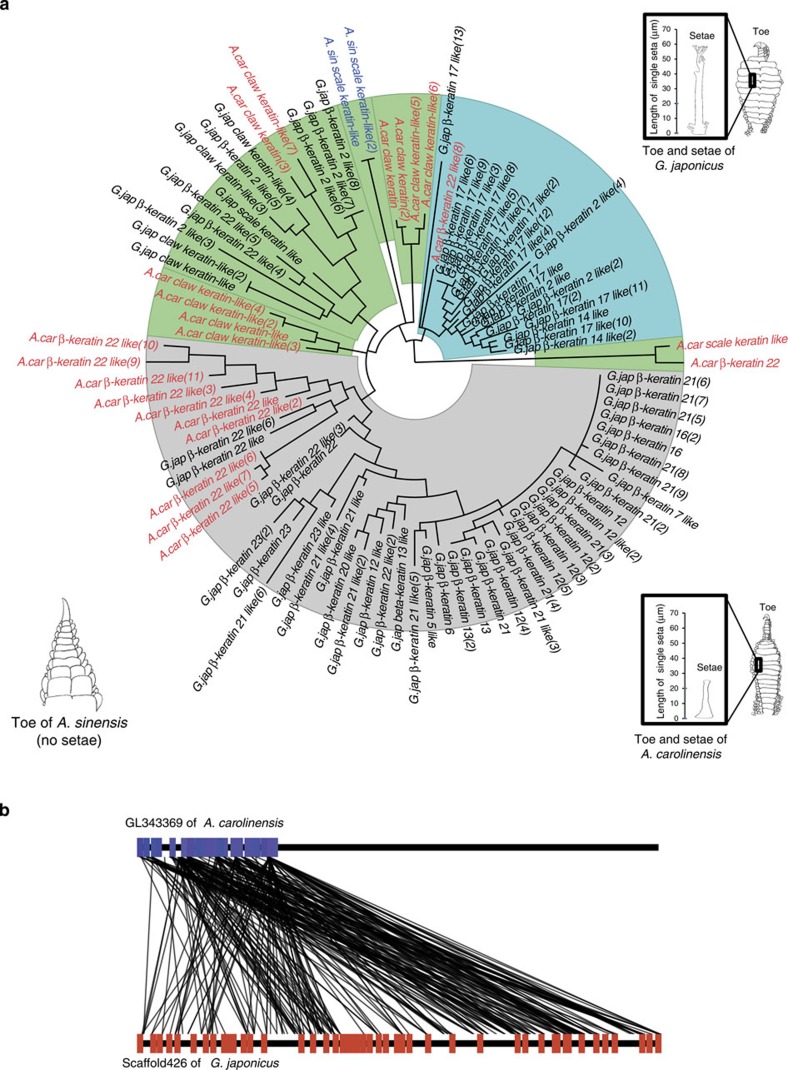
Phylogenetic tree of *β-keratin* families from *G. japonicus*, *An. carolinensis* and *Al. sinensis*. (**a**) The *β-keratins* in black font belong to *G. japonicus*, those in red belong to *An. carolinensis* and those in blue belong to *Al. sinensis*. Gene copy number is listed in parentheses. The green background denotes β-keratins in scales and claws. The grey background denotes β-keratins in setae. The blue background denotes β-keratins in digital scales and pad lamella for supporting setae. A schematic diagram of toe of *G. japonicus*, *An. carolinensis* and *Al. sinensis*, which possess branched setae, unbranched setae and no setae are presented, respectively. The setae of gecko *G. japonicus* are ∼60 μm in length, and that in *An. carolinensis* is ∼25 μm. (**b**) Synteny diagram of *β-keratin* genes in *A. carolinensis* (upper line: GL343369, blue: 23 *β-keratin* genes) and *G. japonicus* (lower line: scaffold 426, red: 48 *β-keratins*).

**Figure 3:**
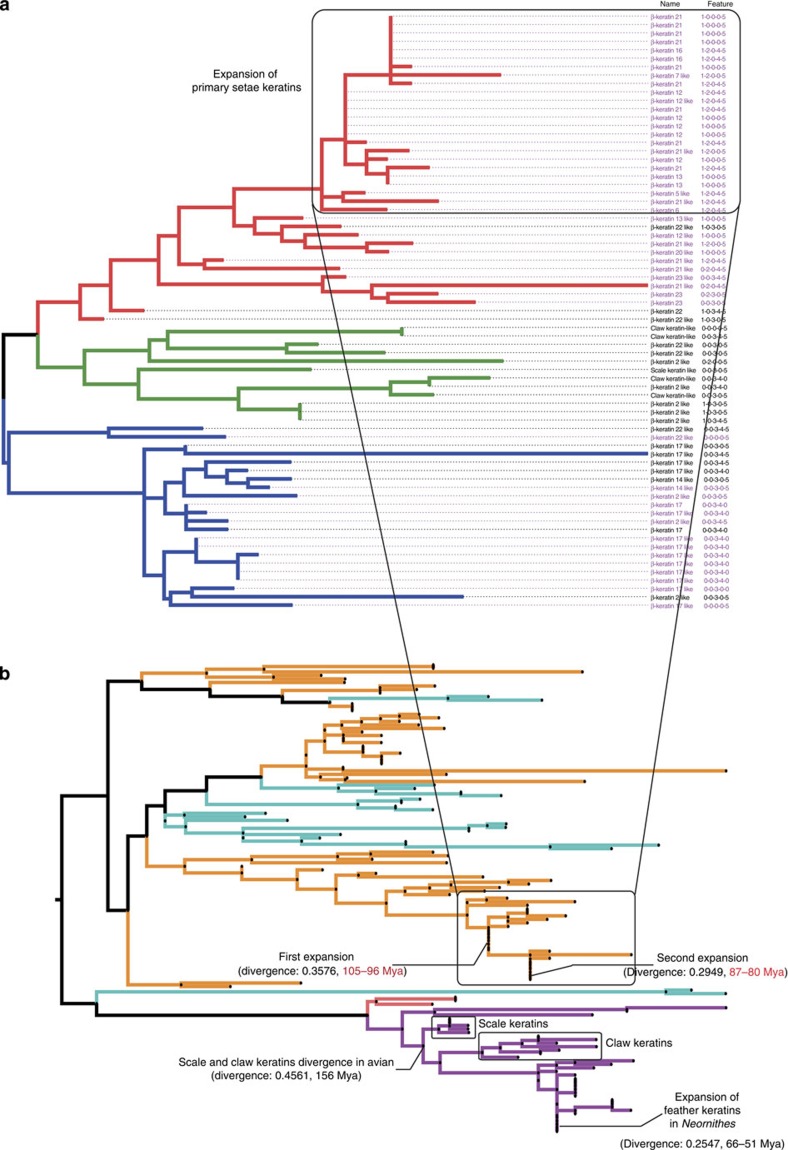
Evolutionary analysis of setae *β*-*keratins*. (**a**) Phylogenetic tree of *β-keratins* from *G. japonicus*. The red branches represent β-keratins belonging to the primary components of setae, the blue branches represent the components in pad lamella for supporting setae and the green branches represent β-keratins in scales or claws. A total of 48 *β-keratins* (purple font) are clustered in scaffold 426, of these 46 have a single exon. The combinatorial numbers following the keratin names indicate the following protein characteristics: 1: S-core box (SEVTIQPPPCTVVVPGPVLA, sequence similarity ≥70%, 35 proteins); 2: cysteine-rich (Cys >10%, 19 proteins); 3: glycine-rich (Gly >15%, 36 proteins); 4: isoelectric point (pI >7, 34 proteins); 5: molecular weight (Wt <15,000, 58 proteins); 0: none of the above. *β-keratins* associated with clinging ability have undergone extensive expansion and have higher isoelectric points. (**b**) Calculation of the expansion period for primary gecko setae *β-keratins* using protein sequences from 71 *β-keratins* of *G. japonicas* (orange), 23 *β-keratins* of *An. carolinensis* (light blue), 2 *β-keratins* of *Al. sinensis* and 1 *β-keratin-*like of *Crocodylus niloticus* (pink), and 36 *β-keratins* associated with bird's claw, scale and feather (violet, including the following birds: *Gallus gallus*, *Chlamydotis macqueenii*, *Opisthocomus hoazin*, *Mesitornis unicolor*, *Haliaeetus leucocephalus*, *Leptosomus discolor*, *Nestor notabilis*, *Chaetura pelagica*, *Pterocles gutturalis*, *Tinamus guttatus*, *Pygoscelis adeliae*, *Tauraco erythrolophus*, *Manacus vitellinus*, *Picoides pubescens*, *Mycteria americana*, *Cathartes aura* and *Eurypyga helias*). The divergence of scale and claw *keratins* occurs in a birds ancestor ∼156 Myr ago, and the feather *keratins* expansion occurred in birds ∼66 Myr ago. *β-keratins* in setae of *G. japonicus* have undergone two expansion periods: one approximately 105–96 Myr ago and the other approximately 87–80 Myr ago.

The emergence of novel lineage-specific morphological features is always accompanied with genes duplications and diversions. A prominent example is the large-scale duplication of *β-keratin* genes that has been crucial to the evolution of scales, claws, beaks and feathers in reptiles and birds. The emergence of setae in geckos also resulted from the duplication and diversion of *β-keratin* genes. The clinging ability of geckos depends primarily on spade-like adhesive setae^21^. Previous reports have indicated that setae are comprised of a corneous material largely made of β-keratins of 8–22 kDa, with cysteine-rich proteins located in setae/spatula, and glycine-rich proteins in the β-layer of epidermis^22^. In addition, β-keratins have high isoelectric points with positive charges that enhance Van der Waals adhesion in the setae. These physicochemical properties indicate that β-keratin proteins are key to the clinging ability of setae^23^. To investigate whether the *β-keratin* genes are associated with varying adhesive ability in different reptile species, we retrieved the *β-keratin* genes from the genomes of *G. japonicus*, *An. carolinensis* and *Al. sinensis*, which possess branched setae, unbranched setae and no setae, respectively. Our analysis showed that the *β-keratin* gene families have undergone major expansion corresponding with adhesive ability (Supplementary Table 19). *G. japonicus*, *An. carolinensis* and *Al. sinensis* contain 71, 23 and 2 *β-keratin* genes, respectively (Fig. 2, Supplementary Table 20). The majority of the extensively expanded families in *G. japonicus* genome contained the setae *β-keratins* genes important for setae production. These β-keratin proteins are usually characterized with S-core box (SEVTIQPPPCTVVVPGPVLA), cysteine-rich and low-molecular weight (∼10 kDa), which had been investigated in *Gekko gecko*. In the *G. japonicus* genome, 35 setae β-keratins with featured S-core box were identified among all 71 β-keratins by aligning them with amino acid sequences of S-core box (sequence similarity ≥70%). Additional important features associated with the above β-keratins, such as cysteine content and molecular weight were summarized in Fig. 3a and Supplementary Table 20. Furthermore, most setae *β-keratins* were clustered on a single scaffold (scaffold 426) of the *G. japonicus* assembly, suggesting regional duplication events. These genomic characteristics may be related to the need for vast abundance of proteins to create the large number of setae in *G. japonicus*. In the other two species, a total of 16 setae *β-keratin* genes were identified in the *An. carolinensis* genome, and no setae *β-keratin* genes were found in the *Al. sinensis* genome. This result suggested that *β-keratin* genes expansion is positively correlated with setae formation in the assessed species. To date the periods of setae *β-keratin* expansion in *G. japonicus*, we established a phylogenetic tree using 133 β-keratin protein sequences from several reptiles and birds^24^, and calculated the divergence value of the branch site in the tree. The timescale of *β-keratin* expansion was determined by reference to the timescale of divergence of scale and claw β-keratins in birds (∼156 Myr ago) and the period of feather keratins expansion (66–51 Myr ago)^25^,^26^. The results showed that setae β-keratins experienced twice expansions: one at 105–96 Myr ago and the other at 87–80 Myr ago (Fig. 3b). The expansion period of setae *β-keratin* genes proposed by this strategy was very close to the period of setae emergence in gecko indicated by fossil evidence^27^. Of course, the comparative analysis of *β-keratin* genes expansion is based on only three genomes, and it is not possible to conclude whether the patterns seen in the *G. japonicus* genome are unique to this species.

### Nocturnal vision adaptation in *G. japonicus*

As a nocturnal animal, *G. japonicus* possesses several sensory system characteristics, such as light sensitivity^28^, reduced colour vision, multifocal optical system, high olfaction^29^ and special auditory senses^30^. Collectively, these features improve the ability of *G. japonicus* to catch prey, evade predators and communicate in low-light environments^28^.

**Figure 4:**
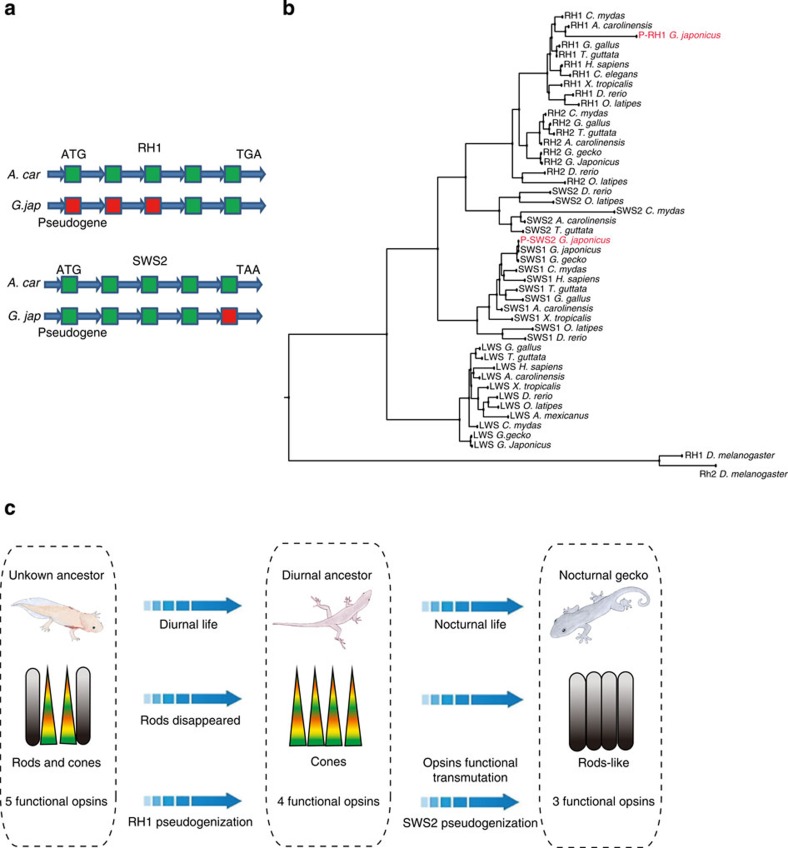
*Opsins* genes in the *G. japonicus* genome. (**a**) Analysis of *RH1* and *SWS2* pseudogenes showed mutations in both initiation and termination codons. In addition, the exons of *RH1* and *SWS2* in *G. japonicus* were lost, incomplete or shifted in comparison with those in *A. carolinensis*. The green boxes indicate exons with similarity to those in *A. carolinensis*. The red boxes represent the lost exons. (**b**) Phylogenetic analysis of *opsin* genes from *Drosophila melanogaster*, *Danio rerio*, *Oryzias latipes*, *Xenopus tropicalis*, *Chelonia mydas*, *Gallus gallus*, *Taeniopygia guttata*, *Anolis carolinensis*, *Gekko gecko* and *Homo sapiens*. The pseudogenes in *G. japonicus* (*P-RH1* and *P-SWS2*) are indicated in red. (**c**) *Opsin* evolution in *G. japonicus* at genomic level agrees with observed evolutionary variation of retinal cell type and visual sense.

Vertebrate photoreceptor cells are categorized as rods and cones. Rods are responsible for dim-light vision, and cones for daylight and colour vision. Most geckos are nocturnal and possess retinas primarily made up of single and double cones^31^. A premise termed the transmutation theory, proposes that cones in nocturnal geckos were transformed from the cones of some ancestral diurnal lizard^32^. We assessed our genome data for evidence indicating how retinal pigment genes evolved during scotopic adaptation. The photosensitive molecules within photoreceptor cells consist of chromophore and opsin (protein moiety). The visual opsins are classified into five paralogs: rod pigments *RH1* (rhodopsin), cone pigments *RH2* (*RH1*-like), *SWS1* (short wavelength-sensitive type 1), *SWS2* (*SWS1*-like) and *LWS*/*MWS* (long wavelength-sensitive and middle wavelength-sensitive). We identified nine *opsin* genes in the genome of *G. japonicus* and 20 *opsin* genes in the genome of *A. carolinensis* (Supplementary Tables 21 and 22), a diurnal lizard with typical tetra chromatic visual system. Our analysis revealed that *A. carolinensis* possessed a complete set of *opsin* paralogs, while *G. japonicus* had only three functional *opsins*: *SWS1*, *LWS* and *RH2*, all of which are usually found in cones (Supplementary Table 22). In searching for the remaining two opsins, we were able to identify the rod pigment *RH1* and the cone pigment *SWS2* in *G. japonicus*, however, they were nonfunctional pseudogenes. The phylogenetic relationship between the opsins form different species is shown in Fig. 4b. We found that *RH1* was more divergent between *G. japonicus* and *A. carolinensis* than was *SWS2*, indicating that the loss of *RH1* occurred earlier than that of *SWS2* (Fig. 4a). These results were in agreement with the hypothesis that the ancestors of modern geckos are diurnal lizard without rod opsins^28^. These ancestral species later lost the cone *opsin SWS2*, potentially as a partial adaptation towards nocturnal vision^33^. Our data provide, for the first time, the evidence of a *RH1* deficiency at the genome level in nocturnal geckos.

These data lead to the question of what functional opsins are responsible for nocturnal vision in *G. japonicus*. Previous reports have suggested that opsin *RH2* amino acid site 89 is an important functional determinant of rod and cone visual pigments^33^. Our comparative analysis indicated that *RH2* in *G. japonicus* possesses the amino acid replacements F89C, which enables *RH2* to have rod pigment-specific biochemical characteristics^32^,^33^,^34^ (Supplementary Table 23). Our results suggest a possible functional switch or compensatory change in cone pigment of gecko that allows them to receive more light. In conclusion, our results surrounding the evolution of opsin genes at genomic level support the hypothesis that nocturnal *G. japonicus* evolved from the diurnal ancestors. Furthermore, the pseudogenization and functional switch of *opsins* is concordant with the variation of retinal cell type, which includes the loss of rods and transformation of cones (Fig. 4c). One thing to note here is that the evolution pattern of *opsin* we proposed is based on genome data of few species. More data, including genomes of other related species, and phylogenetic analyses are necessary to test this hypothesis and to determine how and when particular changes occurred in the *opsin* families.

### Diversified olfaction of *G. japonicus*

**Figure 5:**
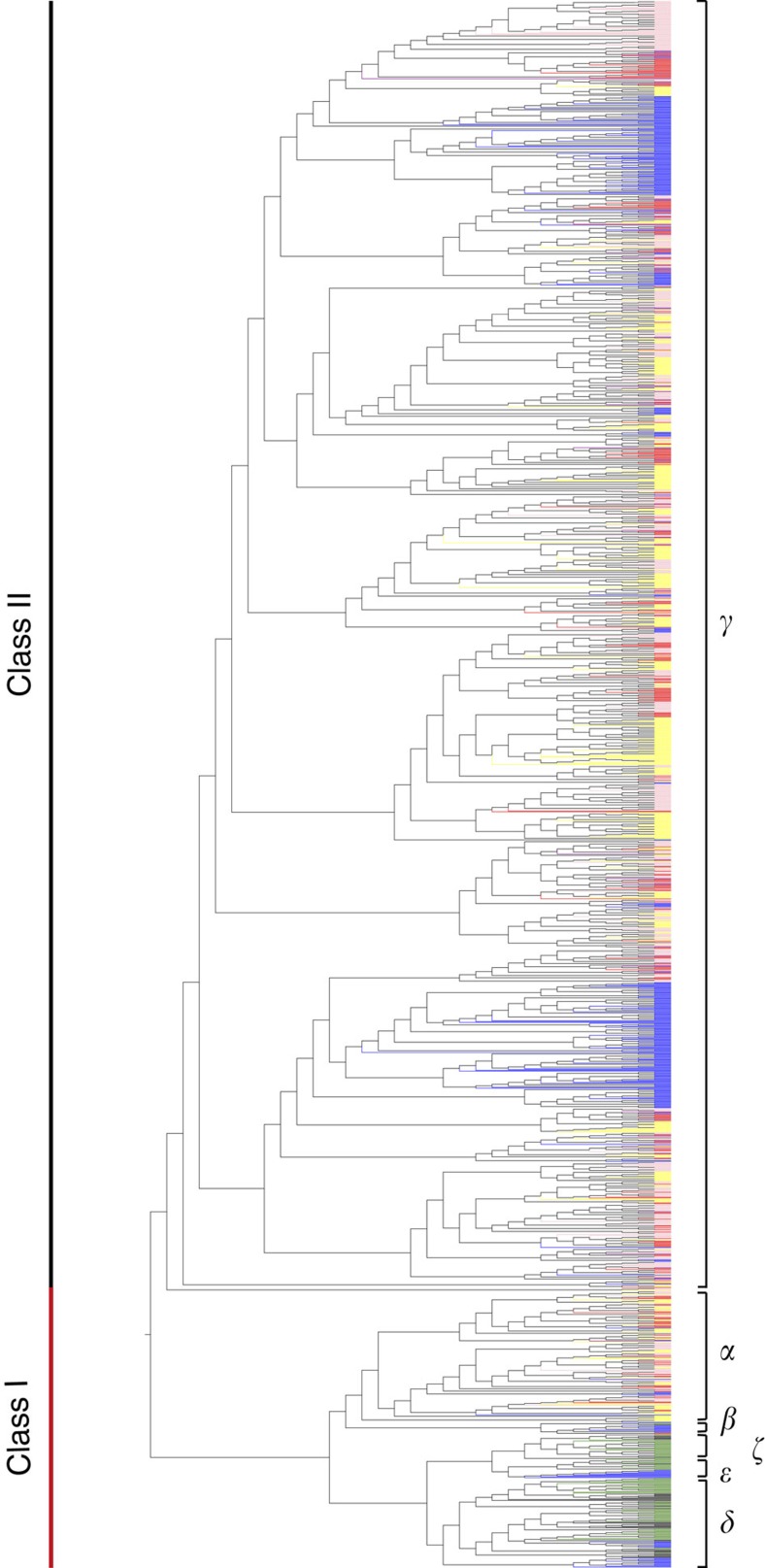
Phylogenetic tree of functional *OR* genes in seven species. The Class I genes (including *α*, *β*, *ɛ*, *ζ* and *δ OR*s) encode proteins used for scent detection in water. The Class II gene (*γ*-*OR*) encode proteins used for scent detectionin air. *G. japonicus* has undergone extensive expansion in Class II genes. Red, *G. japonicus*; blue, *X. tropicalis*; green, *D. rerio*; purple, *An. carolinensis*; yellow, *H. sapiens*; pink, *Al. sinensis*; black, *T. rubripes*.

Improved survival in a low-light environment typically includes the evolution of more sensitive sense of smell to better obtain food and safety^35^. Our analysis of expanded gene families in 16 species revealed that *G. japonicus* had a significant expansion of olfactory receptor (OR) genes. *OR* genes are divided into Class I and Class II. The Class I genes encode odour receptors that detect molecules in water, and can be divided into the following sub-groups: *α*, *β*, *ɛ*, *ζ* and *δ*. The class II genes encode odour receptors that detect molecules in air, and only have a *γ* sub-group^36^. According to our gene family analysis, *G. japonicus* displayed more diversity of *OR* genes (*α-*, *β-* and *γ*-ORs) than any of other assessed reptiles and more than even human (*α-*, *γ*-ORs). Usually, a single air OR can only identify one or a few scents^37^, thus, the presence of a higher number of ORs may indicate greater diversity in odour sensing. To investigate the types and numbers of ORs in species with different features, ORs from seven species (*G. japonicus*, *X. tropicalis*, *D. rerio*, *An. carolinensis*, *H. sapiens*, *Al. sinensis* and *T. rubripes*) were compared. Because the assembly of ORs is difficult, then the assembly quality of these genomes is a notable concern for the comparative analysis of ORs. The parameters associated with the genome assembly quality are listed in Supplementary Table 24. The comparative results show that the number of OR genes in *G. japonicus* (251) is almost three times that in *A. carolinensis*, a diurnal relative of *G. japonicus* (87) (Fig. 5, Supplementary Table 25). Furthermore, nocturnal *G. japonicus* had many functional γ-ORs for detecting airborne chemicals, which may serve to complement visual senses when seeking prey^33^,^38^. The ability to detect airborne chemicals has been shown to contribute to survival in nocturnal lizards and birds^38^,^39^. Likewise, the substantial expansion of *OR* genes for scenting airborne odorants in *geckos* might improve their nighttime survival.

### Positive selection for tail regeneration in *G. japonicus*

**Figure 6:**
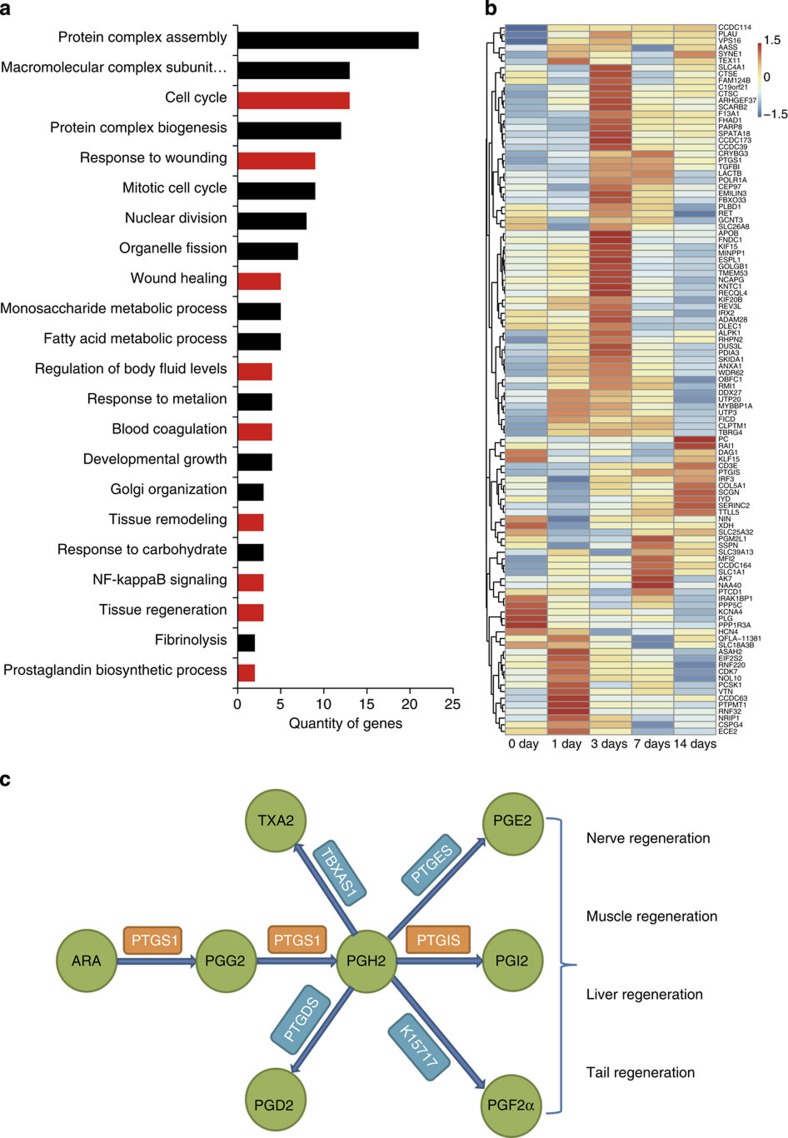
Positively selected genes (PSGs) related to tail regeneration in *G. japonicus*. (**a**) Analysis of 155 PSGs in *G. japonicus* based on representative gene ontology biological processes. Categories with red bars such as cell cycle (GO:0007049), response to wounding (GO:0009611), wound healing (GO:0042060), tissue regeneration (GO:0042246), tissue remodelling (GO:0048771), blood coagulation (GO:0007596) and prostaglandin biosynthetic process (GO:0001516) are likely to be involved in tail regeneration after autotomy. (**b**) Heatmap of 107 PSGs in *G. japonicus* at different time points following tail autotomy. Approximately 70% of the PSGs were detected in transcriptome data. (**c**) Pathway of arachidonic acid (ARA) metabolism in *G. japonicus*. The circles with green backgrounds represent ARA and its derivatives. The boxes with orange backgrounds show the key synthases under positive selection in *G. japonicus* genome. The boxes with blue backgrounds show the normal synthases in *G. japonicus* genome.

Geckos can detach their tails when they are attacked, and this adaptive physiological process has evolved in many saurian animals to enable quick escape from predators. On tail detachment, multiple tissue regeneration pathways initiate some conserved repair processes including wound healing^40^, blastema formation and tissue remodelling^41^,^42^,^43^. Following this, a new tail will grow within a few months. We searched the *G. japonicus* genome for genomic regions showing positive selection for tail regeneration in *G. japonicus*, as such genes are likely important to this process. To identify PSGs, single-copy orthologs were selected from six reptile gene families (*G. japonicus*, *An. carolinensis*, *Al. sinensis*, *C. mydas*, *Pelodiscus sinensis* and *P. bivittatus*). Then, we carried out multiple alignments of the single-copy orthologs and calculated the Ka/Ks ratios to identify PSGs in three reptile species (*G. japonicus* and *An. carolinensis* two species with tail regeneration ability, as well as *Al. sinensis*, a species without this ability). We obtained 155, 178 and 171 PSGs from *G. japonicus*, *An. carolinensis* and *Al. sinensis*, respectively (Supplementary Table 26). Gene Ontology annotation revealed that some PSGs of *G. japonicus* were likely related to regenerative abilities, as these PSGs were enriched in the categories of wound healing, tissue regeneration, cell proliferation or migration, prostaglandin biosynthetic process and other relevant categories (Fig. 6a). These biological processes are essential for successful regeneration, as reported by many studies on limb and tail regeneration in other species^40^,^41^,^42^,^43^,^44^. The PSGs in *A. carolinensis* and *G. japonicus* contain several shared Gene Ontology terms, such as cell proliferation and prostaglandin biosynthetic process. It is notable that prostacyclin synthase (*PTGIS*) and prostaglandin–endoperoxide synthase 1 (*PTGS1*), which are involved in prostaglandin biosynthesis, were under positive selection in both *G. japonicus* and *A. carolinensis.* This indicates the likelihood that these genes are involved in similar biological processes that may promote tail regeneration after injury. To investigate whether the PSGs in *G. japonicus* had activity during tail regeneration, we collected transcriptome data from regenerating stump tissue at different time points following tail amputation. The results showed that 69% of the PSGs were upregulated at 1 day, 3 days or 7 days after autotomy (Fig. 6b). Among these genes, the expression of both *PTGIS* and *PTGS1*, which encode key enzymes in prostaglandin synthesis, increased by 3 days after tail amputation. Prostanoids have been reported to be involved in the regeneration of various tissues and organs, including liver, muscle, nerve and tail in different species^45^,^46^,^47^,^48^ (Fig. 6c). These findings support the hypothesis that PSGs may be involved in tail regeneration or other adaptive physiological processes in *G. japonicus.*

## Discussion

Gecko is an important clade of reptile that possesses several amazing abilities, such as the ability to cling at vertical surface and tail regeneration. These characteristics have been extensively investigated for many years. However, the absence of genomic data for Gekkonidae species hinders the mechanism study underlying these interesting phenomena. In this study we acquired the genome sequence of *G. japonicus* and used this information to investigate the genetic basis of many behavioural and physiological characteristics of geckos by searching for key genes potentially involved in clinging ability, low-light visual activity, highly developed olfaction and regenerative ability. Although our study cannot be considered as an in-depth analysis at the present stage, it provides a foundation for future mechanistic studies, particularly with regard to regeneration. We identified potential candidate genes that might contribute to the regeneration process using genome comparative analysis. Such candidates include *PTGIS* and *PTGS1*, which are involved in prostaglandin synthesis. Prostaglandin metabolism was recently reported to be tightly associated with regeneration in multiple tissues^49^. The timing and manner of the involvement of these genes in tail regeneration of *G. japonicus* is an attractive topic for further study. In addition, gecko species can also serve as an important model for the studies of sex determination^50^ and reproductive strategies^51^, for the reasons that these species situate on the nodes switching environment sex determination to genetic sex determination, oviparity pattern to viviparity pattern. The *G. japonicus* genomic data obtained in the current study will be of great value in studying these essential evolutionary events.

## Methods

### *G. japonicus* sample

The *G. japonicus* used in this study were sampled in Jiangsu, China. The adult geckos were freely fed mealworms and given water during the whole experiment. All experimental protocols pertinent to animals were given before approval by the Laboratory Animal Care and Use Committee of the Nantong University.

### Genome sequencing and assembly

Genomic DNA was extracted from tissues of an adult male *G. japonicus*. The DNA was fragmented and the fragments were purified by electrophoresis for whole-genome sequencing. DNA Libraries were constructed according to the ‘*Mate Pair Library v2 Sample Preparation Guide for 2*–*5 kb Libraries*' and ‘*Paired-End Sample Preparation Guide*' from Illumina. PCR amplification was performed following the addition of adaptors, and the products were clustered for the mate-pair libraries (insert size ≥2 Kb). The genomic DNA was sequenced using Illumina Hiseq2000 (20 lanes, 330.90 Gb, 131.35 × ). Raw data were generated, after filtering 233.49 Gb of clean data remained for *de novo* assembly. Whole-genome assembly was performed using SOAPdenovo^52^ and SSPACE software^53^. Furthermore, 10 fosmid clones were subjected to Sanger sequencing and were used as reference data to ensure genomic coverage.

### Genome annotation and evolution

Repeats in DNA sequence of *G. japonicus* were characterized by homologue-based identification using RepeatMasker (http://www.repeatmasker.org) and Repbase^54^. Repeated proteins were marked using RepeatProteinMask(http://www.repeatmasker.org), and *de novo* interspersed repeat annotation was performed using RepeatModeler (http://www.repeatmasker.org/RepeatModeler.html). An extra RepeatMasker analysis was applied after *de novo* identifications of repeats. In addition, tandem repeats were identified using Tandem Repeat Finder^55^. Gene prediction was created by GLEAN (http://sourceforge.net/projects/glean-gene) integration of *de novo* and homologous gene models. RNA-seq data were subsequently used to refine the gene set. *De novo* prediction was performed based on the repeat-masked genome. Two programs AUGUSTUS^56^ and GENSCAN^57^ were applied in the prediction. The homologue-based prediction included the mapping of protein sequences (downloaded from NCBI) of closely related representative species *An. carolinensis*, *Gallus gallus*, *Homo sapiens*, *Meleagris gallopavo* and *Xenopus tropicalis* to the genome using TblastN, aligning and searching for accurate spliced alignments using GeneWise^58^. The EST of *G. japonicus* (downloaded from NCBI) was aligned against the assembled genome using BLAT to generate spliced alignments, and PASA was used to filter the overlapping sequences to link the spliced alignments and predict the possible gene model. Evidences were integrated by GLEAN to produce a consensus gene set. In addition, transcriptomes of multiple tissues were aligned to the genome using TopHat^59^. These were assembled using Cufflinks^60^ to improve the accuracy and completeness of the predicted gene set. To conclude gene functions, we scanned the final gene set with KEGG^61^, SwissProtand TrEMBL^62^. InterProScan was also used to confirm motifs and domains in the final gene set of *G. japonicus*^63^.

### Gene family expansion

We identified gene family expansion and contraction using CAFÉ^64^, which employed a random birth and death model to study gene gain and loss in gene families across a user-specified phylogeny. The global parameter *λ*, which described both the gene birth (*λ*) and death (*μ*=−*λ*) rate across all branches in the tree for all gene families, was estimated using maximum likelihood. A conditional *P* value was calculated for each gene family, and families with conditional *P* values less than threshold (0.0001) were considered to have accelerated rates of gain or loss. We identified branches that were responsible for low overall *P* values of significant families.

### Phylogenetic analyses

We constructed a phylogenetic tree that included *Alligator sinensis*, *Anolis carolinensis*, *Pelodiscus sinensis*, *Chelonia mydas*, *Xenopus tropicalis*, *Canis familiaris*, *Oryzias latipes*, *Homo sapiens*, *Meleagris gallopavo*, *Gallus gallus*, *Ornithorhynchus anatinus*, *Danio rerio*, *Taeniopygia guttata*, *Python molurus bivittatus* and *G. japonicus* using 696 single-copy orthologous genes from gene family construction. Each of the orthologous genes was subjected to multiple sequence alignment with Muscle^65^ and concatenated into a super sequence. PhyML^66^ was used to construct the phylogenetic tree under the GTR and Inv-γ model functions. The same sequence set was applied to estimate the periods of species divergence using the program PAML MCMCTREE under correlated molecular clock function in the approximate likelihood calculation method^67^. The correlated molecular clock and REV substitution model were selected to perform estimation. The MCMC process of PAML mcmctree was run to sample 100,000 times with a sample frequency of 50, after a burn-in of 5,000,000 iterations. Fossil calibrates were derived from http://www.fossilrecord.net/dateaclade/index.html.

### Positively selected genes

We calculated Ka/Ks ratios for all single-copy orthologs of *G. japonicus*, *Anolis carolinensis*, *Alligator sinensis*, *Python molurus bivittatus*, *Chelonia mydas* and *Pelodiscus sinensis.* Alignment quality was essential for estimating positive selection. Thus, orthologous genes were first aligned using PRANK^68^, a favored alignment tool for molecular evolution studies^69^. We used Gblocks to remove ambiguously aligned blocks within the PRANK alignments^70^. We employed ‘codeml' in the PAML package with the free-ratio model to estimate Ka, Ks and Ka/Ks ratios on different branches. The differences in the mean Ka/Ks ratios for single-copy genes between *G. japonicus* and each of the other species were compared with paired Wilcoxon rank-sum tests. After filtering out the false positive, we obtained a final total of 155 positive selection genes in G*. japonicus*.

## Additional information

**Accession codes:** This Whole Genome Shotgun project of *Gekko japonicus* has been deposited at DDBJ/EMBL/GenBank under the accession LNDG00000000. The version described in this paper is version LNDG01000000. The RNA-Seq data of *Gekko japonicus* have been deposited in NCBI database under the accession code SRA304902.

**How to cite this article:** Liu, Y. *et al.* *Gekko japonicus* genome reveals evolution of adhesive toe pads and tail regeneration. *Nat. Commun.* 6:10033 doi: 10.1038/ncomms10033 (2015).

## Supplementary Material

Supplementary Information Supplementary Figures 1-11, Supplementary Tables 1-26, Supplementary Methods and Supplementary References

## References

[b1] Wang D. Y., Kumar S. & Hedges S. B. Divergence time estimates for the early history of animal phyla and the origin of plants, animals and fungi. Proc. Biol. Sci. 266, 163–171 (1999) . 10097391 10.1098/rspb.1999.0617 PMC1689654

[b2] Peter U. The original descriptions of reptiles. Zootaxa 2334, 59–68 (2010) .

[b3] Torstrom S. M., Pangle K. L. & Swanson B. J. Shedding subspecies: the influence of genetics on reptile subspecies taxonomy. Mol. Phylogenet. Evol. 76, 134–143 (2014) . 24662681 10.1016/j.ympev.2014.03.011

[b4] Vidal N. & Hedges S. B. The phylogeny of squamate reptiles (lizards, snakes, and amphisbaenians) inferred from nine nuclear protein-coding genes. C. R. Biol. 328, 1000–1008 (2005) . 16286089 10.1016/j.crvi.2005.10.001

[b5] Wever E. G. The lizard ear: Gekkonidae. J. Morphol. 143, 121–165 (1974) . 4834697 10.1002/jmor.1051430202

[b6] Fleming P. A., Valentine L. E. & Bateman P. W. Telling tails: selective pressures acting on investment in lizard tails. Physiol. Biochem. Zool. 86, 645–658 (2013) . 24241062 10.1086/673864

[b7] Autumn K. *et al.* Adhesive force of a single gecko foot-hair. Nature 405, 681–685 (2000) . 10864324 10.1038/35015073

[b8] Autumn K. *et al.* Evidence for van der Waals adhesion in gecko setae. Proc. Natl Acad. Sci. USA 99, 12252–12256 (2002) . 12198184 10.1073/pnas.192252799 PMC129431

[b9] McLean K. E. & Vickaryous M. K. A novel amniote model of epimorphic regeneration: the leopard gecko, Eublepharis macularius. BMC Dev. Biol. 11, 50 (2011) . 21846350 10.1186/1471-213X-11-50 PMC3180301

[b10] Boesel L. F., Greiner C., Arzt E. & del Campo A. Gecko-inspired surfaces: a path to strong and reversible dry adhesives. Adv. Mater. 22, 2125–2137 (2010) . 20349430 10.1002/adma.200903200

[b11] Castoe T. A. *et al.* The Burmese python genome reveals the molecular basis for extreme adaptation in snakes. Proc. Natl Acad. Sci. USA 110, 20645–20650 (2013) . 24297902 10.1073/pnas.1314475110 PMC3870669

[b12] Wan Q. H. *et al.* Genome analysis and signature discovery for diving and sensory properties of the endangered Chinese alligator. Cell Res. 23, 1091–1105 (2013) . 23917531 10.1038/cr.2013.104 PMC3760627

[b13] Wang Z. *et al.* The draft genomes of soft-shell turtle and green sea turtle yield insights into the development and evolution of the turtle-specific body plan. Nat. Genet. 45, 701–706 (2013) . 23624526 10.1038/ng.2615 PMC4000948

[b14] Alfoldi J. *et al.* The genome of the green anole lizard and a comparative analysis with birds and mammals. Nature 477, 587–591 (2011) . 21881562 10.1038/nature10390 PMC3184186

[b15] Green R. E. *et al.* Three crocodilian genomes reveal ancestral patterns of evolution among archosaurs. Science 346, 1254449 (2014) . 25504731 10.1126/science.1254449 PMC4386873

[b16] Tempel S. Using and understanding RepeatMasker. Methods Mol. Biol. 859, 29–51 (2012) . 22367864 10.1007/978-1-61779-603-6_2

[b17] Daza J. D., Bauer A. M. & Snively E. D. On the fossil record of the Gekkota. Anat. Rec. (Hoboken) 297, 433–462 (2014) . 24482344 10.1002/ar.22856

[b18] Tarling D. H. Gondwanaland, palaeomagnetism and continental drift. Nature 229, 17–21 71 (1971) . 16059055 10.1038/229017a0

[b19] Pyron R. A., Burbrink F. T. & Wiens J. J. A phylogeny and revised classification of Squamata, including 4161 species of lizards and snakes. BMC Evol. Biol. 13, 93 (2013) . 23627680 10.1186/1471-2148-13-93 PMC3682911

[b20] Rodriguez-Marco N. A., Larrea-Goni N. & Solanas-Alava S. Prehistoric eyes: Ophthalmosaurus and the Tuatara. Arch. Soc. Esp. Oftalmol. 88, e33–e34 (2013) . 23623026 10.1016/j.oftal.2012.07.032

[b21] Magin T. M., Vijayaraj P. & Leube R. E. Structural and regulatory functions of keratins. Exp. Cell Res. 313, 2021–2032 (2007) . 17434482 10.1016/j.yexcr.2007.03.005

[b22] Alibardi L. Immunolocalization of keratin-associated beta-proteins (beta-keratins) in pad lamellae of geckos suggest that glycine-cysteine-rich proteins contribute to their flexibility and adhesiveness. J. Exp. Zool. A Ecol. Genet. Physiol. 319, 166–178 (2013) . 23423812 10.1002/jez.1782

[b23] Alibardi L. Cell biology of adhesive setae in gecko lizards. Zoology (Jena) 112, 403–424 (2009) . 19782546 10.1016/j.zool.2009.03.005

[b24] Ye C., Wu X., Yan P. & Amato G. beta-Keratins in crocodiles reveal amino acid homology with avian keratins. Mol. Biol. Rep. 37, 1169–1174 (2010) . 19266314 10.1007/s11033-009-9480-z

[b25] Zhang G. *et al.* Comparative genomics reveals insights into avian genome evolution and adaptation. Science 346, 1311–1320 (2014) . 25504712 10.1126/science.1251385 PMC4390078

[b26] Greenwold M. J. & Sawyer R. H. Linking the molecular evolution of avian beta (beta) keratins to the evolution of feathers. J. Exp. Zool. B Mol. Dev. Evol. 316, 609–616 (2011) . 21898788 10.1002/jez.b.21436

[b27] Arnold E. N & Poinar G. O. A 100 million year old gecko with sophisticated adhesive toe pads, preserved in amber from Myanmar. Zootaxa 1847, 62–68 (2008) .

[b28] Roll B. Gecko vision-visual cells, evolution, and ecological constraints. J. Neurocytol. 29, 471–484 (2000) . 11279363 10.1023/a:1007293511912

[b29] Schwenk K. The evolution of chemoreception in squamate reptiles: a phylogenetic approach. Brain. Behav. Evol. 41, 124–137 (1993) . 8477337 10.1159/000113830

[b30] Bergevin C. Comparison of otoacoustic emissions within gecko subfamilies: morphological implications for auditory function in lizards. J. Assoc. Res. Otolaryngol. 12, 203–217 (2011) . 21136278 10.1007/s10162-010-0253-0 PMC3046335

[b31] Roth L. S., Lundstrom L., Kelber A., Kroger R. H. & Unsbo P. The pupils and optical systems of gecko eyes. J. Vis. 9, 27.1–11 (2009) . 19757966 10.1167/9.3.27

[b32] Kojima D. *et al.* Cone visual pigments are present in gecko rod cells. Proc. Natl Acad. Sci. USA 89, 6841–6845 (1992) . 1379723 10.1073/pnas.89.15.6841 PMC49600

[b33] Yokoyama S. & Blow N. S. Molecular evolution of the cone visual pigments in the pure rod-retina of the nocturnal gecko, Gekko gekko. Gene 276, 117–125 (2001) . 11591478 10.1016/s0378-1119(01)00643-6

[b34] Yokoyama S. & Tada T. Evolutionary dynamics of rhodopsin type 2 opsins in vertebrates. Mol. Biol. Evol. 27, 133–141 (2010) . 19759234 10.1093/molbev/msp217 PMC2794311

[b35] Tonosaki K. & Shibuya T. Action of some drugs on gecko olfactory bulb mitral cell responses to odor stimulation. Brain Res. 167, 180–184 (1979) . 455065 10.1016/0006-8993(79)90275-0

[b36] Niimura Y. Evolutionary dynamics of olfactory receptor genes in chordates: interaction between environments and genomic contents. Hum. Genomics 4, 107–118 (2009) . 20038498 10.1186/1479-7364-4-2-107 PMC3525206

[b37] Hoover K. C. Evolution of olfactory receptors. Methods Mol. Biol. 1003, 241–249 (2013) . 23585047 10.1007/978-1-62703-377-0_18

[b38] Gordon C. E., Dickman C. R. & Thompson M. B. What factors allow opportunistic nocturnal activity in a primarily diurnal desert lizard (Ctenotus pantherinus)? Comp. Biochem. Physiol. A Mol. Integr. Physiol. 156, 255–261 (2010) . 20170741 10.1016/j.cbpa.2010.02.007

[b39] Steiger S. S., Fidler A. E. & Kempenaers B. Evidence for increased olfactory receptor gene repertoire size in two nocturnal bird species with well-developed olfactory ability. BMC Evol. Biol. 9, 117 (2009) . 19467156 10.1186/1471-2148-9-117 PMC2701422

[b40] Hutchins E. D. *et al.* Transcriptomic analysis of tail regeneration in the lizard Anolis carolinensis reveals activation of conserved vertebrate developmental and repair mechanisms. PLoS ONE 9, e105004 (2014) . 25140675 10.1371/journal.pone.0105004 PMC4139331

[b41] Delorme S. L., Lungu I. M. & Vickaryous M. K. Scar-free wound healing and regeneration following tail loss in the leopard gecko, Eublepharis macularius. Anat. Rec. (Hoboken) 295, 1575–1595 (2012) . 22933425 10.1002/ar.22490

[b42] Love N. R. *et al.* Amputation-induced reactive oxygen species are required for successful Xenopus tadpole tail regeneration. Nat. Cell Biol. 15, 222–228 (2013) . 23314862 10.1038/ncb2659 PMC3728553

[b43] Niethammer P., Grabher C., Look A. T. & Mitchison T. J. A tissue-scale gradient of hydrogen peroxide mediates rapid wound detection in zebrafish. Nature 459, 996–999 (2009) . 19494811 10.1038/nature08119 PMC2803098

[b44] Kimura Y. *et al.* Expression of complement 3 and complement 5 in newt limb and lens regeneration. J. Immunol. 170, 2331–2339 (2003) . 12594255 10.4049/jimmunol.170.5.2331

[b45] Rudnick D. A., Perlmutter D. H. & Muglia L. J. Prostaglandins are required for CREB activation and cellular proliferation during liver regeneration. Proc. Natl Acad. Sci. USA 98, 8885–8890 (2001) . 11447268 10.1073/pnas.151217998 PMC37530

[b46] Hsueh Y. C., Wu J. M., Yu C. K., Wu K. K. & Hsieh P. C. Prostaglandin E(2) promotes post-infarction cardiomyocyte replenishment by endogenous stem cells. EMBO Mol. Med. 6, 496–503 (2014) . 24448489 10.1002/emmm.201303687 PMC3992076

[b47] Kogawa S., Yasuda H., Terada M., Maeda K. & Kikkawa R. Apoptosis and impaired axonal regeneration of sensory neurons after nerve crush in diabetic rats. Neuroreport 11, 663–667 (2000) . 10757497 10.1097/00001756-200003200-00003

[b48] Sharma P. & Suresh S. Influence of COX-2-induced PGE2 on the initiation and progression of tail regeneration in Northern House Gecko, Hemidactylus flaviviridis. Folia Biol. (Praha) 54, 193–201 (2008) . 19393133 10.14712/fb2008054060193

[b49] Zhang Y. *et al.* TISSUE REGENERATION. Inhibition of the prostaglandin-degrading enzyme 15-PGDH potentiates tissue regeneration. Science 348, aaa2340 (2015) . 26068857 10.1126/science.aaa2340 PMC4481126

[b50] Gamble T. A review of sex determining mechanisms in geckos (Gekkota: Squamata). Sex. Dev. 4, 88–103 (2010) . 20234154 10.1159/000289578 PMC2855288

[b51] Pyron R. A. & Burbrink F. T. Early origin of viviparity and multiple reversions to oviparity in squamate reptiles. Ecol. Lett. 17, 13–21 (2014) . 23953272 10.1111/ele.12168

[b52] Li R. *et al.* De novo assembly of human genomes with massively parallel short read sequencing. Genome Res. 20, 265–272 (2010) . 20019144 10.1101/gr.097261.109 PMC2813482

[b53] Boetzer M., Henkel C. V., Jansen H. J., Butler D. & Pirovano W. Scaffolding pre-assembled contigs using SSPACE. Bioinformatics 27, 578–579 (2011) . 21149342 10.1093/bioinformatics/btq683

[b54] Jurka J. *et al.* Repbase Update, a database of eukaryotic repetitive elements. Cytogenet. Genome Res. 110, 462–467 (2005) . 16093699 10.1159/000084979

[b55] Benson G. Tandem repeats finder: a program to analyze DNA sequences. Nucleic Acids Res. 27, 573–580 (1999) . 9862982 10.1093/nar/27.2.573 PMC148217

[b56] Stanke M. & Morgenstern B. AUGUSTUS: a web server for gene prediction in eukaryotes that allows user-defined constraints. Nucleic Acids Res. 33, W465–W467 (2005) . 15980513 10.1093/nar/gki458 PMC1160219

[b57] Burge C. B. & Karlin S. Finding the genes in genomic DNA. Curr. Opin. Struct. Biol. 8, 346–354 (1998) . 9666331 10.1016/s0959-440x(98)80069-9

[b58] Birney E., Clamp M. & Durbin R. GeneWise and Genomewise. Genome Res. 14, 988–995 (2004) . 15123596 10.1101/gr.1865504 PMC479130

[b59] Trapnell C., Pachter L. & Salzberg S. L. TopHat: discovering splice junctions with RNA-Seq. Bioinformatics 25, 1105–1111 (2009) . 19289445 10.1093/bioinformatics/btp120 PMC2672628

[b60] Trapnell C. *et al.* Differential gene and transcript expression analysis of RNA-seq experiments with TopHat and Cufflinks. Nat. Protoc. 7, 562–578 (2012) . 22383036 10.1038/nprot.2012.016 PMC3334321

[b61] Wixon J. & Kell D. The Kyoto encyclopedia of genes and genomes–KEGG. Yeast 17, 48–55 (2000) . 10928937 10.1002/(SICI)1097-0061(200004)17:1<48::AID-YEA2>3.0.CO;2-H PMC2447041

[b62] Boeckmann B. *et al.* The SWISS-PROT protein knowledgebase and its supplement TrEMBL in 2003. Nucleic Acids Res. 31, 365–370 (2003) . 12520024 10.1093/nar/gkg095 PMC165542

[b63] Quevillon E. *et al.* InterProScan: protein domains identifier. Nucleic Acids Res. 33, W116–W120 (2005) . 15980438 10.1093/nar/gki442 PMC1160203

[b64] De Bie T., Cristianini N., Demuth J. P. & Hahn M. W. CAFE: a computational tool for the study of gene family evolution. Bioinformatics 22, 1269–1271 (2006) . 16543274 10.1093/bioinformatics/btl097

[b65] Edgar R. C. MUSCLE: multiple sequence alignment with high accuracy and high throughput. Nucleic Acids Res. 32, 1792–1797 (2004) . 15034147 10.1093/nar/gkh340 PMC390337

[b66] Guindon S. *et al.* New algorithms and methods to estimate maximum-likelihood phylogenies: assessing the performance of PhyML 3.0. Syst. Biol. 59, 307–321 (2010) . 20525638 10.1093/sysbio/syq010

[b67] Yang Z. PAML: a program package for phylogenetic analysis by maximum likelihood. Comput. Appl. Biosci. 13, 555–556 (1997) . 9367129 10.1093/bioinformatics/13.5.555

[b68] Loytynoja A. & Goldman N. Phylogeny-aware gap placement prevents errors in sequence alignment and evolutionary analysis. Science 320, 1632–1635 (2008) . 18566285 10.1126/science.1158395

[b69] Markova-Raina P. & Petrov D. High sensitivity to aligner and high rate of false positives in the estimates of positive selection in the 12 Drosophila genomes. Genome Res. 21, 863–874 (2011) . 21393387 10.1101/gr.115949.110 PMC3106319

[b70] Talavera G. & Castresana J. Improvement of phylogenies after removing divergent and ambiguously aligned blocks from protein sequence alignments. Syst. Biol. 56, 564–577 (2007) . 17654362 10.1080/10635150701472164

