# Supplementary material for: Gekko japonicus genome reveals evolution of adhesive toe pads and tail regeneration
Source: Nat Commun. 2015 Nov 24;6:10033. doi: 10.1038/ncomms10033 (PMC4673495; doi:10.1038/ncomms10033)
Supplement: Supplementary Information — Supplementary Figures 1-11, Supplementary Tables 1-26, Supplementary Methods and Supplementary References [file ncomms10033-s1.pdf]

SUPPLEMENTARY INFORMATION

Supplementary Figures

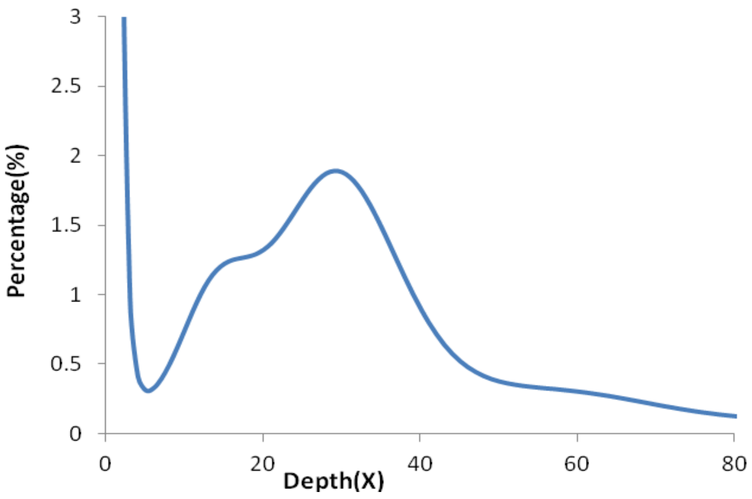

Supplementary Figure 1. Estimation of t *G. japonicus* genome size based on 17-mer analysis. Depth distribution of 17-mers. The abscissa represent sequencing depth and the vertical ordinate represent percentage, calculated as the K-mer number under different depths divided by the K-mer total number.

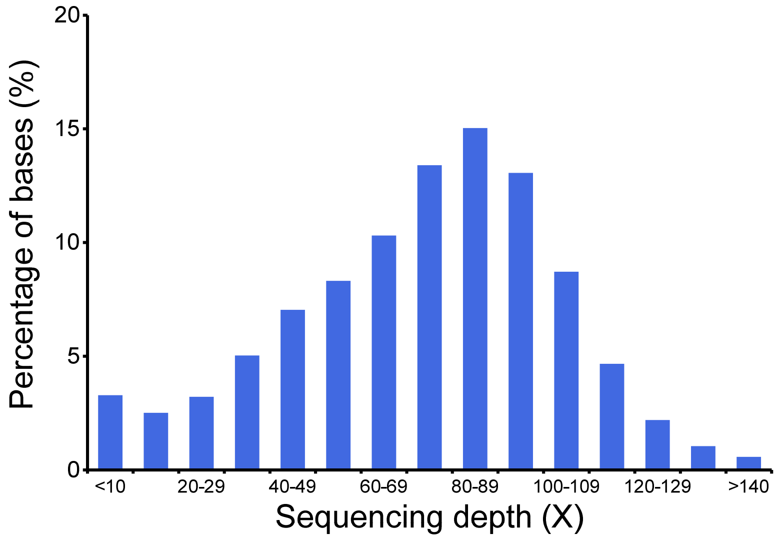

Supplementary Figure 2. Distribution of sequencing depths. The abscissa represents the sequencing depths and the ordinate represents the percentages of bases (percentage = base number at a given sequencing depth / total base number).

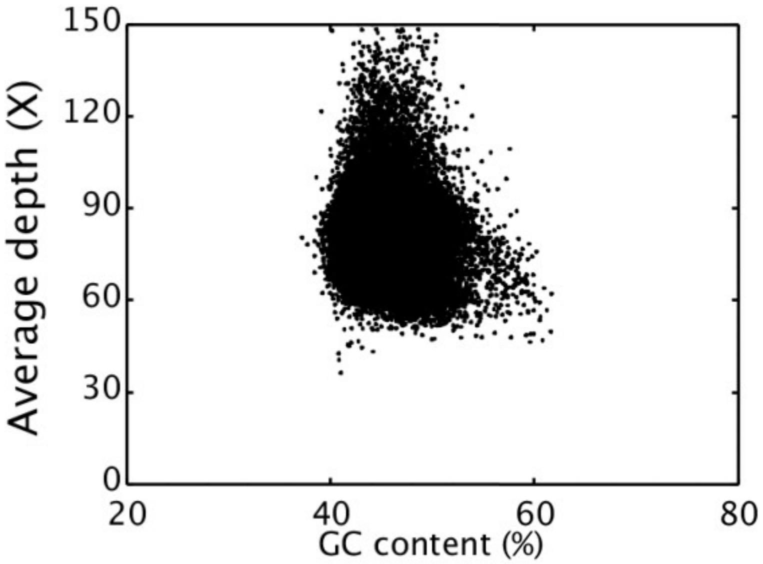

Supplementary Figure 3. Distribution diagram of GC content and sequencing depths. The abscissa represents GC content and the ordinate represents average sequencing depth. The computing window equals 50 kb without overlap.

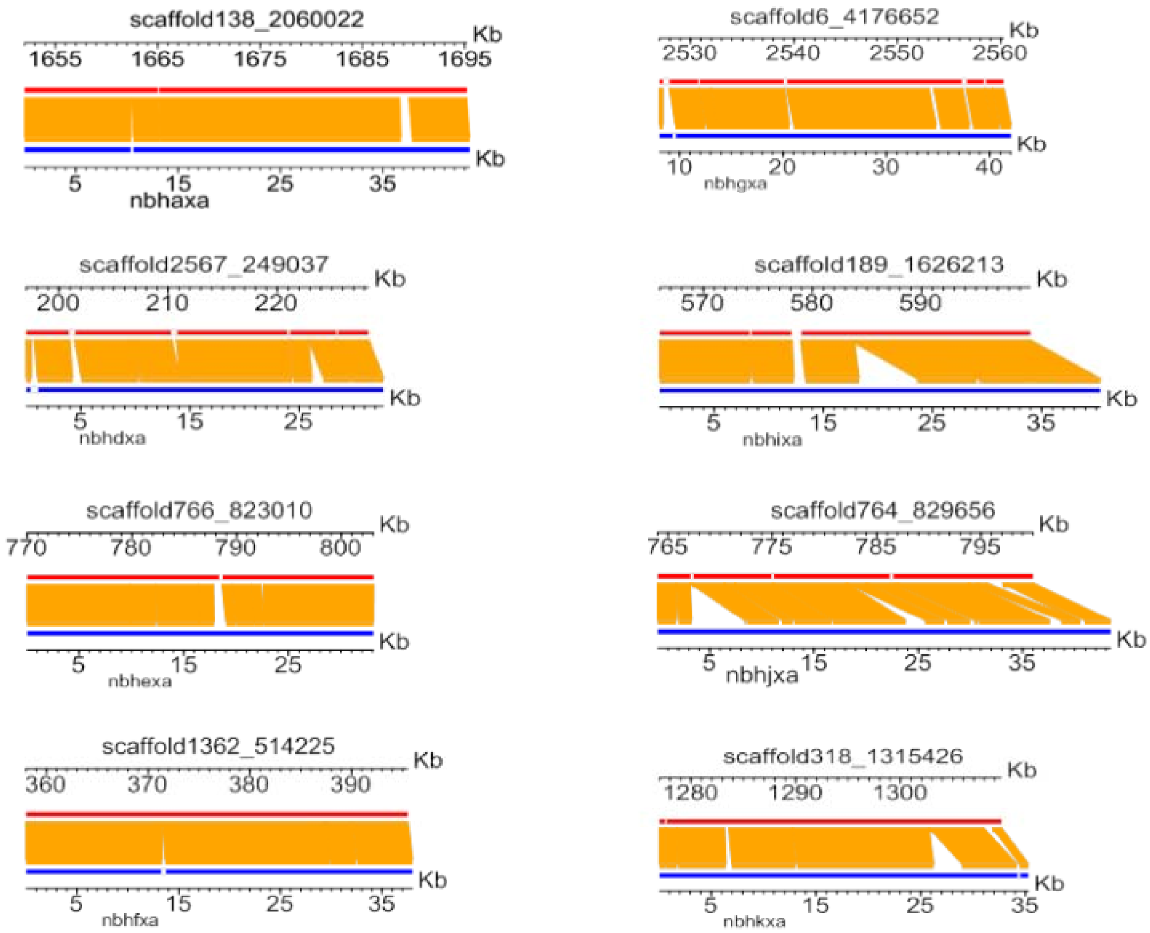

Supplementary Figure 4. Sequence alignments of fosmid clones and assembled scaffolds. The red bars denote scaffold sequences and the blue bars denote fosmid clone sequences. The yellow polygons indicate that the fosmid clone sequences in the indicated regions align very well with the scaffold sequences.

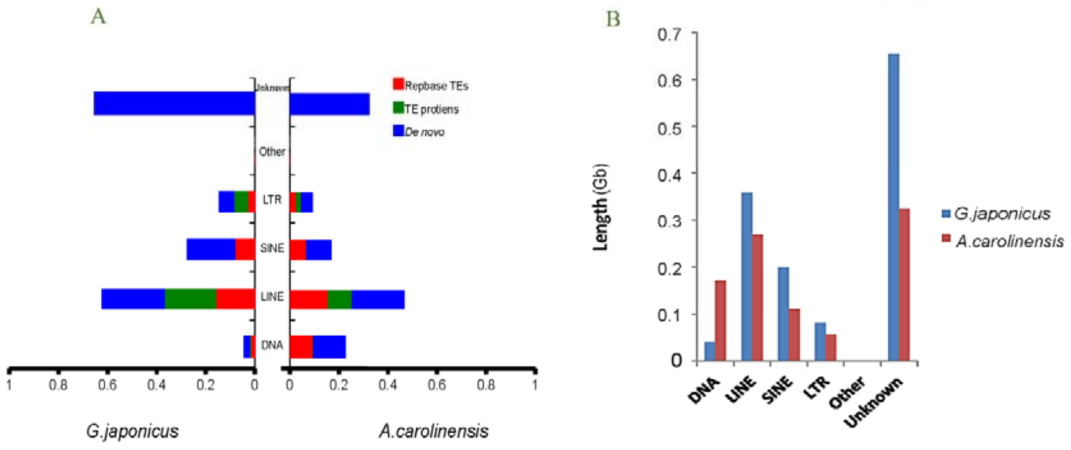

Supplementary Figure 5. Comparison of repeated elements in *G. japonicus* and *A. carolinensis.* (A) Repeats were identified by homology-based and de novo prediction approaches. The homology-based approach identified known repeats with Repbase using RepeatMasker and RepeatProteinMask. *De novo* prediction was performed using RepeatMakser on repeat libraries generated by *RepeatModeler*. Additionally, tandem repeats were searched using program *Tandem Repeats Finder*. **(B)** Comparison of combined repeat sequences from *G. japonicas (G. jap)* and *A. carolinensis (A. car).* Repeats were identified by combined homology-based and de novo approaches.

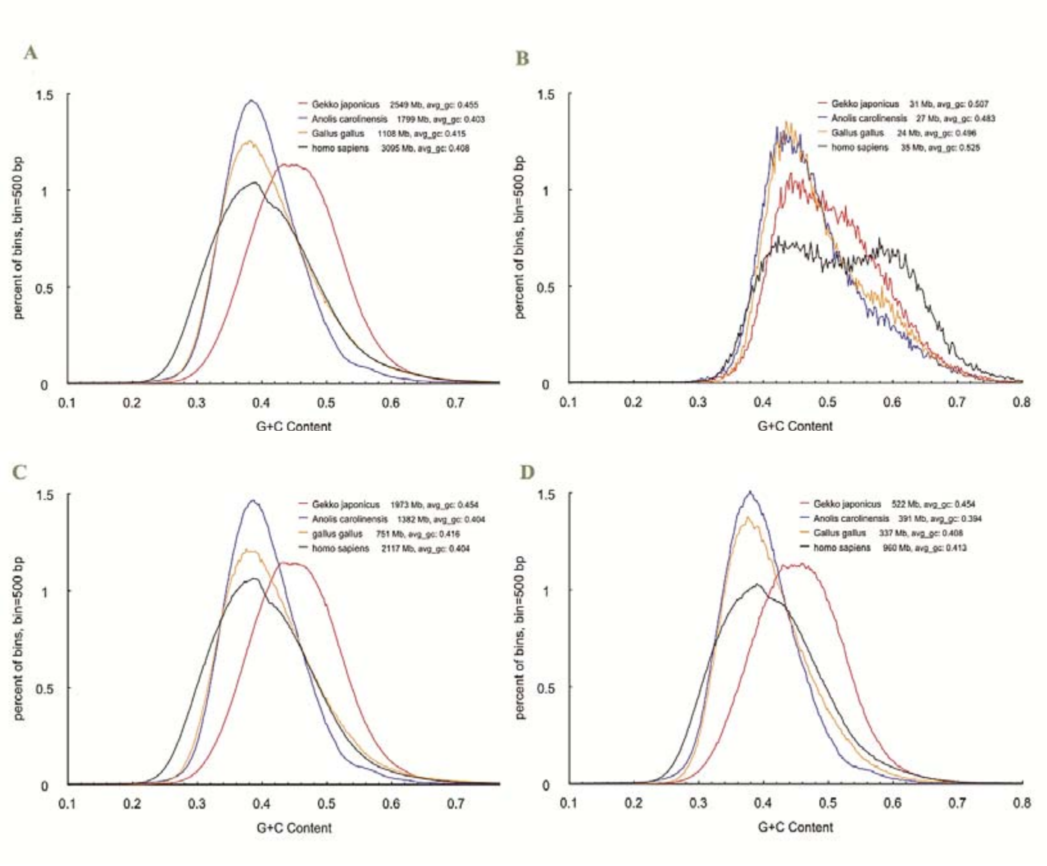

Supplementary Figure 6. GC content in the *Gekko japonicus* genome compared with *Anolis carolinensis, Gallus gallus* and *Homo sapiens* genomes. (A) GC content of whole genomes. (B) GC content of CDS. (C) GC content of intergenic regions; (D) GC content of intron regions. The abscissa represents to GC content and the ordinate represents the bin percentages (bin=500 bp, overlap between two conjoint bins=250 bp)

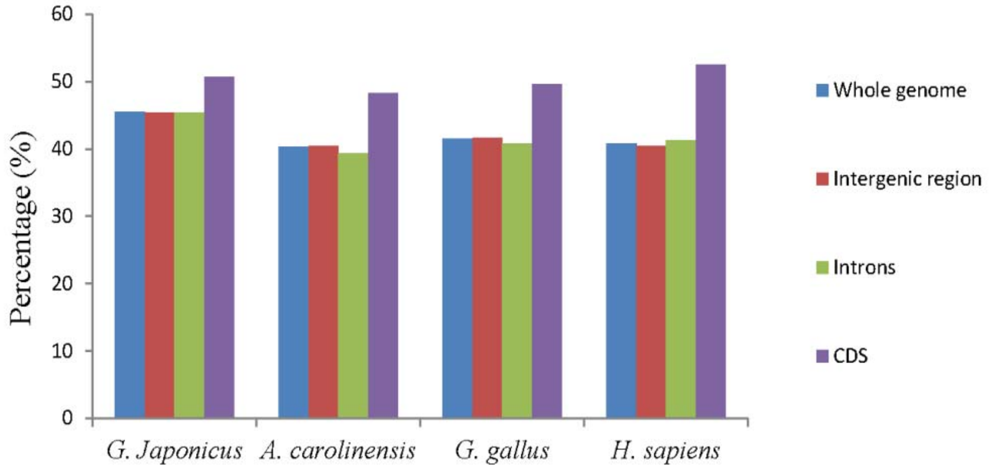

Supplementary Figure 7. Comparison of distributions *G. japonicus, A. carolinensis*, *G. gallus* and *H. sapiens* genomes. The bar diagram shows that the whole genome, intergenic regions and intron regions **GC content** is higher in *G. japonicus* than in other species.

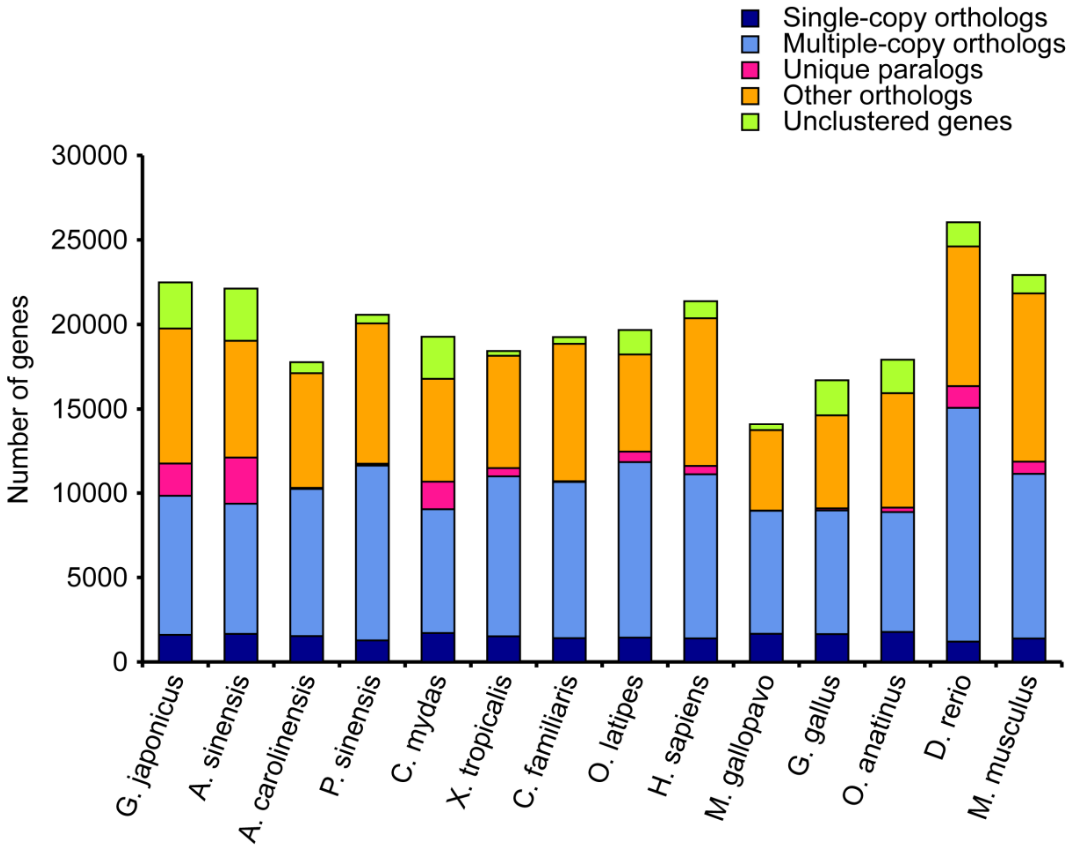

Supplementary Figure 8. Number of orthologous genes in fourteen species. The bar diagram reveals that the highest numbers of unique paralogs exist in the *G. japonicus* and *A. sinensis* genome.

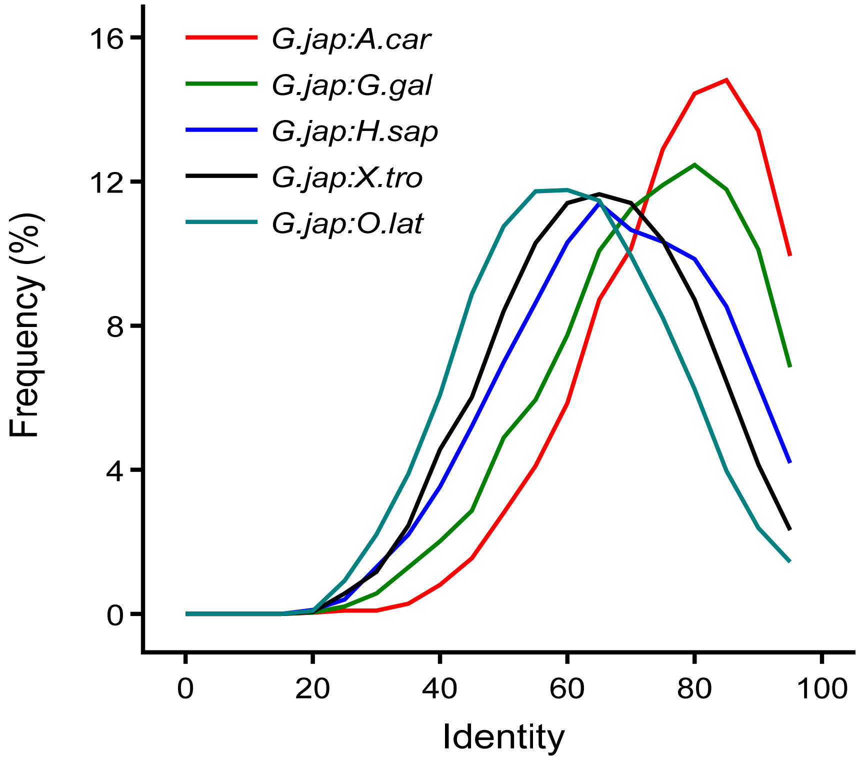

Supplementary Figure 9. Identities of orthologous gene pairs of *G. japonicus* and other species. The abscissa represents identity and the ordinate represnts frequency. *G. jap, Gekko japonicus; A. car, Anolis carolinensis; G. gal, Gallus gallus; H. sap, Homo sapiens; X. tro, Xenopus tropicalis; O. lat, Oryzias latipes*

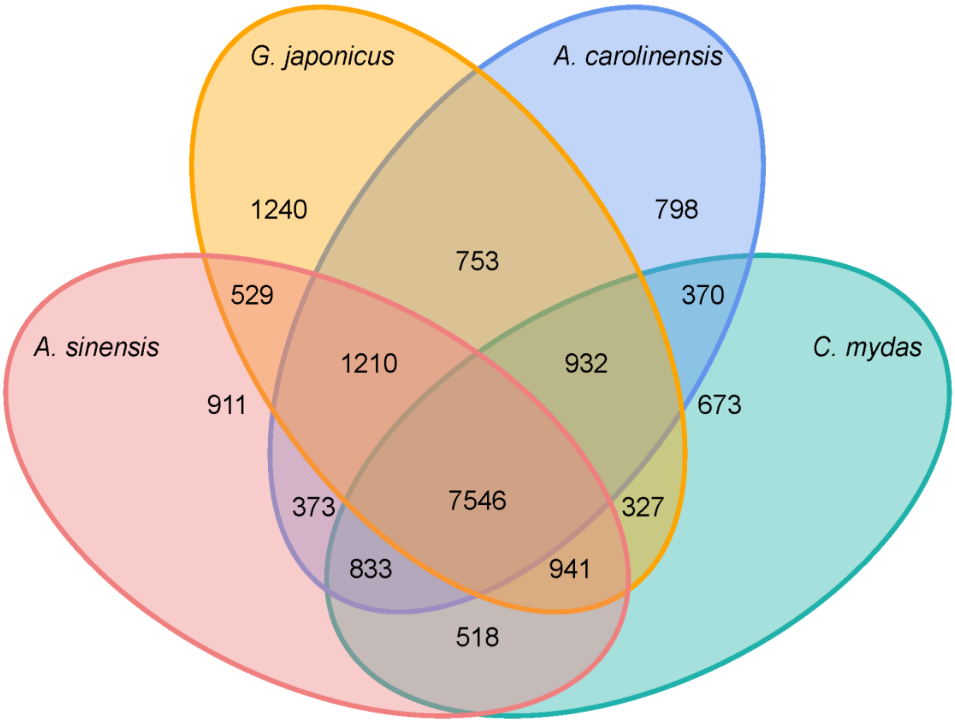

Supplementary Figure 10. Venn diagram of orthologous gene families in four reptiles. Pink, *A. sinensis*; orange, *G. japonicus*; blue, *A. carolinensis*; green, *C. mydas*. The numbers represent quantities of gene families.

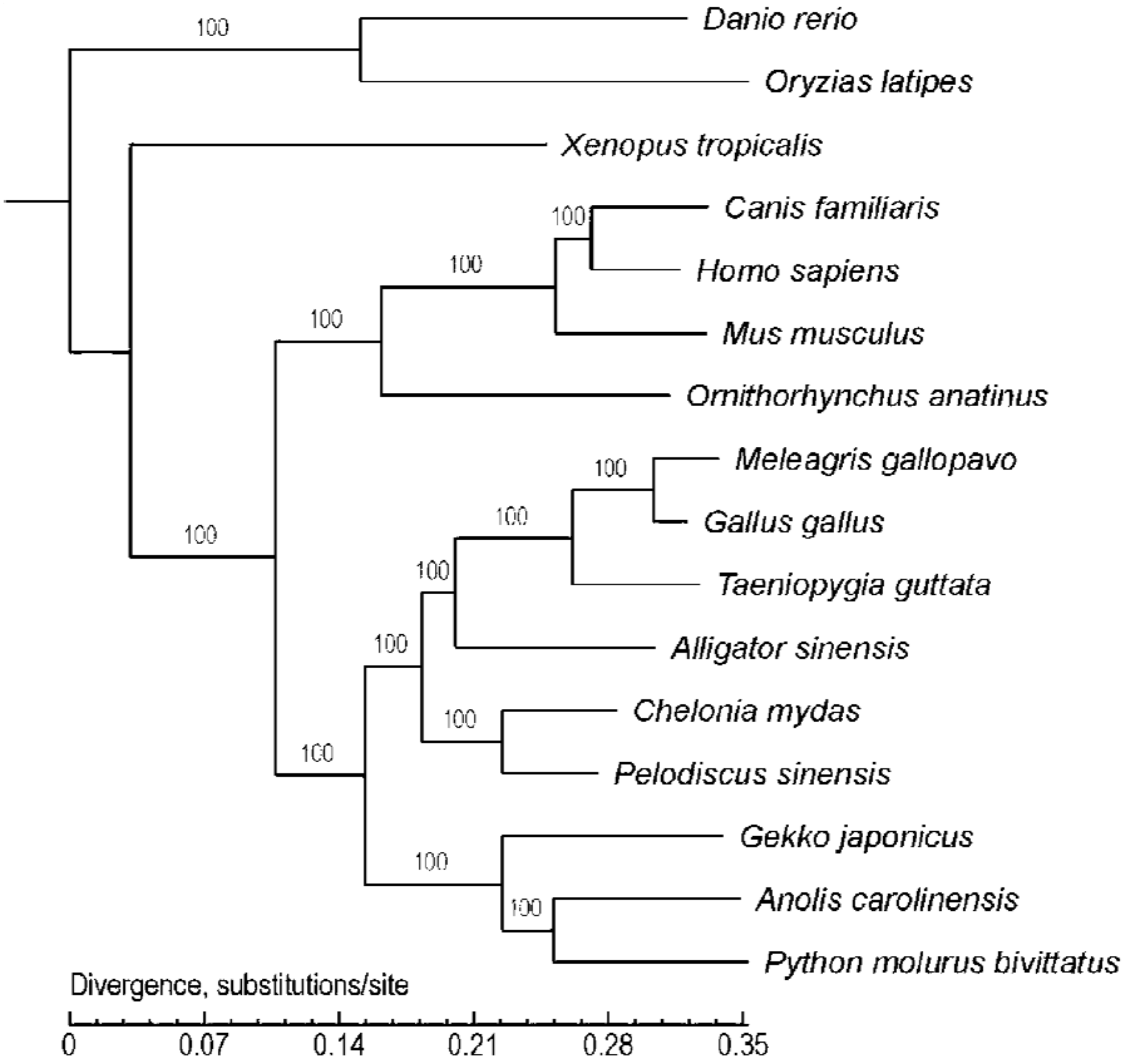

Supplementary Figure 11. Phylogenetic tree constructed using conserved house-keeping proteins from sixteen species. This tree is consistent with the phylogenetic tree constracted using single-copy orthologous genes from gene families.

Supplementary Tables

Supplementary Table 1. Statistic analysis of sequencing data.

	Insert Size	Number of library	Number of lane	Reads Length(bp)	Raw Data			Clean Data		
					Total Data(Gb)	Sequence Depth(X)	Physical Depth(X)	Total Data(Gb)	Sequence Depth(X)	Physical Depth(X)
**Pair-end Libraries**	170bp	2	2	100	69.77	27.69	23.53	65.08	25.83	21.95
	500bp	1	2	100	66.39	26.35	65.86	58.4	23.17	57.94
	800bp	2	3	100	56.26	22.33	89.3	50.51	20.04	80.17
	2Kb	2	4	49	42.52	16.87	344.35	28.32	11.24	229.35
	5Kb	2	2	49	36.07	14.31	730.28	13.3	5.28	269.27
	10Kb	1	3	49	30.11	11.95	1219.23	13.25	5.26	536.52
	20Kb	1	2	49	21.23	8.42	1719.31	3.11	1.23	251.86
	40Kb	1	2	49	8.64	3.43	1399.42	1.52	0.6	246.19
**Total**	----	12	20	----	330.99	131.35	5591.28	233.49	94.65	1693.27

A totally **of** 330.90 Gb raw data with 131.35X genome coverage was obtained from libraries containing various insert sizes. After filtration, 233.49 Gb data remained.

Supplementary Table 2. K-mer analysis of the *G. japonicus* genome.

K	K-mer Number	Peak Depth	Genome Size (bp)	Used Bases	Used Reads	X
17	73,197,410,496	29	2,524,048,637	87,139,774,400	871,397,744	34.5238

Genome size, heterozygosis and repeat content were estimated using K-mer analysis. Reads of 87.14 Gb clean data (34.52X) were split into K-mers, and K-mer depth was computed to obtain depth distribution. The peak K-mer depth is approximately 29, which represents the K-mer expectation depth. Genome size was estimated as K-mer num/Peak depth, which results 2.52 Gb with 660033 scaffolds.

Supplementary Table 3. *G. japonicus* genome assembly.

	Contig		Scaffold	
	**Size(bp)**	**Number**	**Size(bp)**	**Number**
**N90**	3,127	137,292	48,695	5,555
**N80**	7,724	89,575	188,226	3,080
**N70**	11,921	64,171	334,709	2,074
**N60**	16,328	46,567	489,838	1,445
**N50**	21,073	33,312	684,964	1,005
**Longest**	272,958	----	4,762,499	----
**Total Size**	2,460,993,306	----	2,549,379,269	----
**Total Number(>=100bp)**	----	862,142	----	660,033
**Total Number(>=2kb)**	----	157,992	----	17,484

Sizes and numbers of contigs and scaffolds are presented. The data corresponding to fragments shorter than 100 bp are not included.

Supplementary Table 4. Sequencing of fosmid clones from *G. japonicus*.

Fosmid ID	Total Length (bp)	Contig Length (bp)	Average Coverage(X)	Error Rate(%)	Total Reads	Useful Reads	Average Length of Reads(bp)	Scaffold	Contig	Inner Gaps
**Nbhaxa**	43,320	43,320	5.86	0.005	604	423	675.42	1	1	0
**Nbhbxa**	36,983	36,983	6.43	0.037	635	388	647.55	1	1	0
**Nbhdxa**	32,185	32,185	8.16	0.102	845	475	624.29	1	1	0
**Nbhexa**	33,110	33,110	6.36	0.169	603	388	630.15	1	1	0
**Nbhfxa**	37,669	37,342	6.83	0.174	600	437	650.65	1	2	1
**Nbhgxa**	41,874	41,685	6.31	0.03	591	449	656.03	1	2	1
**Nbhhxa**	34,350	34,350	8.03	0.08	598	430	644.29	1	1	0
**Nbhixa**	40,379	40,379	7.42	0.064	600	486	676.47	1	1	0
**Nbhjxa**	43,454	43,454	6.39	0.036	619	458	662.33	1	1	0
**Nbhkxa**	35,192	35,192	6.41	0.378	609	393	575.67	1	2	1

A fosmid library was constructed, and a set of 10 fosmids were sequenced and aligned to the assembled scaffolds for assessing the accuracy of the genome assembly.

Supplementary Table 5. Alignment of fosmid clones and assembled scaffolds.

Fosmid ID	Length (bp)	Coverage Ratio(%)	Alignment Blocks	Aligned Scaffold	Aligned Scaffold Length(bp)	Gap	Gap Length(bp)	Gap Ratio (%)
**nbhaxa**	43,499	99.99	1	1	2,060,022	1	46	0.11
**nbhbxa**	36,983	98.68	356	78	40,038,125	70	36,369	98.34
**nbhdxa**	32,757	100	6	1	249,037	5	1,005	3.07
**nbhexa**	33,110	99.85	7	1	823,010	1	227	0.68
**nbhfxa**	37,996	99.99	2	1	514,225	0	-	0
**nbhgxa**	42,063	100	42	16	12,413,720	7	4,282	10.18
**nbhhxa**	34,350	97.89	296	61	60,964,775	68	23,761	69.17
**nbhixa**	40,379	99.99	6	1	1,626,213	3	814	2.02
**nbhjxa**	43,454	100	14	1	829,656	5	479	1.1
**nbhkxa**	35,292	99.8	5	1	1,315,426	1	33	0.09

Ten fosmid clones were used as reference data, and the assembled genome sequence was aligned with them (BLASTn, E-value threshold was 1e-5) to assess the coverage rate. The alignments were contiguous and of high quality, and the average coverage rate of the fosmid clones was approxmately 99.65%.

Supplementary Table 6. Alignment of EST and unigenes with assembled scaffolds.

Dataset			Number	Total length (bp)	Covered by assembly (%)	with >90% sequence in one scaffold		with >50% sequence in one scaffold	
						Number	Percent (%)	Number	Percent (%)
EST		all	6,957	4,144,080	93.26	5,698	81.90	6,161	88.56
		>200bp	6,870	4,130,502	93.51	5,658	82.36	6,100	88.79
		>500bp	5,786	3,759,633	96.02	4,905	84.77	5,262	90.94
Transcriptome	0d	all	59,216	27,605,888	99.25	53,701	90.69	57,858	97.71
		>200bp	47,067	25,538,187	99.34	42,844	91.03	45,897	97.51
		>500bp	14,336	15,424,923	99.69	12,628	88.09	13,797	96.24
		>1000bp	5,255	9,215,029	99.75	4,447	84.62	4,975	94.67
	1d	all	65,468	29,083,775	99.04	59,377	90.70	63,797	97.45
		>200bp	52,913	26,907,600	99.16	48,141	90.98	51,500	97.33
		>500bp	14,861	15,522,777	99.56	13,037	87.73	14,252	95.90
		>1000bp	5,187	8,879,014	99.69	4,347	83.81	4,910	94.66
	1w	all	62,357	29,712,135	99.14	56,384	90.42	60,772	97.46
		>200bp	50,085	27,613,733	99.23	45,443	90.73	48,730	97.29
		>500bp	15,965	17,123,884	99.64	14,040	87.94	15,362	96.22
		>1000bp	6,046	10,295,741	99.80	5,155	85.26	5,759	95.25
	2w	all	61,023	28,847,852	99.15	55,180	90.42	59,527	97.55
		>200bp	47,805	26,596,763	99.26	43,329	90.64	46,553	97.38
		>500bp	15,399	16,580,293	99.68	13,496	87.64	14,825	96.27
		>1000bp	5,654	9,904,643	99.82	4,742	83.87	5,373	95.03
	all transcriptome	all	61,280	40,007,826	99.04	53,786	87.77	59,107	96.45
		>200bp	61,280	40,007,826	99.04	53,786	87.77	59,107	96.45
		>500bp	23,330	28,296,026	99.58	19,675	84.33	22,237	95.32
		>1000bp	10,285	19,219,765	99.72	8,314	80.84	9,696	94.27

The EST of *Gekko japonicus* was downloaded from NCBI. The Illumina RNA-Seq data were assembled into unigenes using Trinity, and redundant data were removed using TGICL. The EST and the unigenes built by Trinity were aligned on the assembled scaffolds using BLAT with an identity cutoff of 90%.

Supplementary Table 7. Annotation of repetitive sequence.

Method type	Repeat Size(bp)	Percentage in genome(%)
**TRF**	70,455,156	2.76
**RepeatMasker**	283,308,240	11.11
**RepeatProteinMask**	265,473,979	10.41
**De novo**	1,173,572,719	46.03
**Total**	1,247,674,559	48.94

Tandem Repeat Finder (TRF) software was used to find tandem repeat sequences from whole genome sequences; RepeatMasker and RepeatProteinMask were used to find transposable elements based on RepBase; *De novo* data were derived from the sequence library based on RepeatModeler and then annotated by RepeatMasker; The total indicates the non-redundant repeat library from the results of all four methods.

Supplementary Table 8. Annotation of assorted transposable element (TE) repetitive sequences.

	RepBase TEs		TE Proteins		De novo		Combined TEs	
	Length (bp)	% in Genome	Length (bp)	% in Genome	Length (bp)	% in Genome	Length (bp)	% in Genome
**DNA**	17,212,398	0.68	3,467,050	0.14	24,871,577	0.98	39,761,136	1.56
**LINE**	159,723,212	6.27	208,009,527	8.16	256,595,560	10.07	357,416,401	14.02
**LTR**	29,548,410	1.16	54,236,195	2.13	63,268,398	2.48	80,920,638	3.17
**SINE**	80,561,573	3.16	0	0	197,223,912	7.74	199,653,476	7.83
**Other**	16,730	0	0	0	0	0	16,730	0
**Unknown**	0	0	0	0	655,154,086	25.7	655,154,086	25.7
**Total**	283,308,240	11.11	265,473,979	10.41	1,170,537,428	45.91	1,224,093,478	48.02

The RepBase TEs and TE proteins were both based on RepBase, but they were annotated using RepeatMasker and RepeatProteinMask, respectively. *De novo* data were derived from the sequence library based on RepeatModeler and then annotated using RepeatMasker. The combined TEs represent the non-redundant results from the three methods.

Supplementary Table 9. Comparison of TE repetitive sequences in *Gekko japonicus, Alligator sinensis, Anolis carolinensis, Chelonia mydas, Python_molurus_bivittatus* and *Pelodiscus sinensis.*

Type	*Pelodiscus sinensis*		*Gekko japonicus*		*Chelonia mydas*	
	**Length (bp)**	**% in genome**	**Length (bp)**	**% in genome**	**Length (bp)**	**% in genome**
**DNA**	88,713,876	4.013585	39,761,136	1.55964	78,842,269	3.525822
**LINE**	255,255,299	11.548236	357,416,401	14.019742	266,402,552	11.913509
**SINE**	28,764,535	1.301362	199,653,476	7.831454	48,829,888	2.18367
**LTR**	34,341,121	1.553658	80,920,638	3.174131	57,312,680	2.56302
**Other**	4,276	0.000193	16,730	0.000656	2,668	0.000119
**Unknown**	564,130,422	25.522335	655,154,086	25.698573	418,136,924	18.699062
**Total**	902,500,973	40.830863	1,224,093,478	48.015354	809,606,231	36.20555

Type	*Anolis carolinensis*		*Alligator sinensis*		*Python molurus bivittatus*	
	**Length (bp)**	**% in genome**	**Length (bp)**	**% in genome**	**Length (bp)**	**% in genome**
**DNA**	171,235,648	9.517711	45,457,729	1.998261	44,266,185	3.084677
**LINE**	268,654,704	14.932509	662,766,423	29.134326	174,321,941	12.147578
**SINE**	110,540,272	6.144108	18,148,171	0.797769	26,382,999	1.838492
**LTR**	56,066,445	3.116315	234,171,367	10.29386	12,828,899	0.893978
**Other**	541,026	0.030072	4,480	0.000197	18,287	0.001274
**Unknown**	324,060,470	18.012102	49,115,746	2.159063	155,204,443	10.81538
**Total**	817,348,789	45.430316	862,821,730	37.92849	399,417,756	27.83332

The *Gekko japonicus* genome contains more TE repetitive sequences than the other species listed.

Supplementary Table 10. Comparison of repeat subtypes between *G. japonicus* and *A. carolinensis* based on *de novo* prediction and Repbase library.

A. Statistics of repeat at sub-family level based on *de novo* prediction (Top 10)

Species	*G. japonicus*			*A. carolinensis*		
TE/Class	Copy Number	Base	Genome (%)	Copy Number	Base	Genome (%)
SINE/MIR	540,822	100,065,945	3.925	86,860	12,027,539	0.472
LINE/CR1	375,589	100,935,809	3.959	206,613	62,190,685	2.439
LINE/L2	279,901	71,136,771	2.79	189,614	54,840,384	2.151
SINE/5S	203,670	29,852,875	1.171	44,128	5,906,803	0.232
SINE/Sauria	199,286	44,238,986	1.735	378,330	81,213,083	3.186
LINE/RTE	136,547	29,071,034	1.14	140,719	22,964,166	0.901
SINE/ID	134,887	21,444,087	0.841	-	-	-
DNA/hAT	108,618	19,401,168	0.761	228,518	44,751,353	1.755
LINE/L1	90,013	43,459,934	1.705	40,470	18,481,849	0.725
LTR/Gypsy	49,953	37,304,715	1.463	17,930	34,791,707	1.365

B. Statistics of repeat at sub-family class level based on Repbase library (Top 10)

Species	*G. japonicus*			*A. carolinensis*		
TE/Class	Copy Number	Base	Genome (%)	Copy Number	Base	Genome (%)
LINE/CR1	361,593	85,639,631	3.359	148,306	49,622,712	1.946
SINE/MIR	251,477	30,667,465	1.203	14,571	1,190,599	0.047
SINE/Sauria	132,606	33,827,644	1.327	352,350	62,934,667	2.469
LINE/RTE	122,591	44,701,031	1.753	73,625	24,406,676	0.957
SINE/5S	119,846	10,232,328	0.401	19,817	1,874,766	0.074
DNA/hAT	105,730	9,025,548	0.354	317,367	37,713,763	1.479
LINE/L2	76,473	16,255,958	0.638	76,083	15,651,502	0.614
SINE/Deu	60,770	4,432,580	0.174	965	81,286	0.003
LTR/Gypsy	53,387	17,736,470	0.696	62,824	15,974,828	0.627
LINE/L1	43,050	8,860,810	0.348	19,169	4,326,305	0.17

The data reveal that *G. japonicus* had much higher SINE/MIR, LINE/CR1, SINE/5S and LINE/L1 than *A. carolinensis, while A. carolinensis* had much higher SINE/Sauria and DNA/hAT than *G. japonicus.*

Supplementary Table 11. Comparison of segment duplications between *G. japonicus* and *A. carolinensis*.

	G. japonicus			*A. carolinensis*		
**Cutoff (bp)**	**Block**	**Median size (bp)**	**Genome coverage (bp)**	**Block**	**Median size (bp)**	**Genome coverage (bp)**
1,000	39,863	1,294	52,772,568	11,719	1,734	42,990,491

Over 39,863 duplicate blocks longer than 1Kb are present in the *G. japonicus* genome, and 11,719 are present in *A. carolinensis* genome.

Supplementary Table 12. Comparison of repeat sequences between *G. japonicus* and *A. carolinensis.*

		G. japonicus			*A. carolinensis*		
		**Length(bp)**	**In genome(%)**	**GC(%)**	**Length(bp)**	**In genome(%)**	**GC(%)**
**All**	**mRNA**	557,912,812	21.88	45.77	418,426,099	23.26	40.08
	**Exon**	30,467,763	1.2	50.77	27,243,149	1.51	48.31
	**Intron**	505,855,676	19.84	45.49	391,203,310	21.74	39.51
**Ortholog**	**mRNA**	401,277,801	15.74	45.56	295,652,515	16.43	39.98
	**Exon**	21,077,160	0.83	50.58	19,763,511	1.1	48.47
	**Intron**	368,844,589	14.47	45.3	275,904,526	15.34	39.38
**Repeat sequence**		1,247,674,559	48.94	-	831,410,597	46.21	-

Collectively, the above data indicate that the larger genome size of *G. japonicus* might be primarily due to the greater abundance of repeat sequences compared with *A. carolinensis* (the repeat sequences in *G. japonicus* produced 417 Mb more data than those in *A. carolinensis*).

Supplementary Table 13. Final gene set for *G. japonicus*.

Gene set		Number	Average gene length (bp)	Average CDS length (bp)	Average exon per gene	Average exon length (bp)	Average intron length (bp)
De novo	***AUGUSTUS***	39,519.00	31,222.50	1,017.02	5.88	172.95	6,189.29
	GENSCAN	22,464.00	29,493.60	1,798.39	9.9	181.71	3,112.86
Homolog	*A. carolinensis*	22,364.00	21,555.97	1,189.39	6.69	177.86	3,581.06
	G. gallus	19,740.00	24,079.51	1,197.55	6.97	171.89	3,834.90
	H. sapiens	21,170.00	24,138.91	1,244.50	7	177.83	3,816.82
	M. gallopavo	19,391.00	23,169.66	1,196.08	6.93	172.51	3,703.39
	X. tropicalis	20,222.00	22,444.66	1,206.38	6.73	179.35	3,708.81
EST		90,216.00	6,594.65	1,178.87	2.36	498.6	3,969.40
RNA-Seq		23,795.00	23,173.16	1,926.18	7.33	262.91	3,358.52
Final gene set		22,487.00	25,675.78	2,425.75	7.9	306.91	3,367.67

A total of 22,487 genes were finally obtained, with an average exons number per gene of 7.90, an average gene length of 25,676 bp, and an average CDS length of 2,426 bp.

Supplementary Table 14. PHC evidence analysis of the GLEAN gene set of *G. japonicus.*

	>=20% overlap		>=50% overlap		>=80% overlap	
	**No.**	**Ratio(%)**	**No.**	**Ratio(%)**	**No.**	**Ratio(%)**
**P(single)**	564	2.51	915	4.07	2,974	13.23
**P(more)**	1,182	5.26	2,008	8.93	2,905	12.92
**H(single)**	345	1.53	105	0.47	151	0.67
**H(more)**	18	0.08	398	1.77	713	3.17
**C(single)**	238	1.06	305	1.36	480	2.13
**C(more)**	10,203	45.37	9,344	41.55	6,630	29.49
**P+H**	397	1.77	594	2.64	912	4.06
**P+C**	153	0.68	196	0.87	466	2.07
**H+C**	6,456	28.71	5,588	24.85	3501	15.57
**P+H+C**	564	2.51	915	4.07	2,974	13.23

P represents the evidence from *de novo* forecasted data. C represents the evidence from cDNA/EST forecasted data. H stands for the evidence from homologous forecasted data. “Single” indicates only one piece of data was used as evidence. “More” indicates that multiple types of data were used as evidence. Percentage of overlap represents the ratio of overlap in CDS areas between these predictions and the final gene set.

Supplementary Table 15. Annotation of ncRNA in the *G. japonicus* genome.

Type		Copy	Average length (bp)	Total length (bp)	Genome(%)
	miRNA	445	90.89	40,445	0.001586
	tRNA	226	79.96	18,071	0.000709
rRNA	rRNA	79	109.08	8,617	0.000338
	18S	45	109.44	4,925	0.000193
	28S	34	108.59	3,692	0.000145
	5.8S	0	0	0	0
	5S	0	0	0	0
snRNA	snRNA	552	127.41	70,333	0.002759
	CD-box	131	94.89	12,430	0.000488
	HACA-box	87	146.31	12,729	0.000499
	splicing	319	135.13	43,106	0.001691

The results indicate that numerous ncRNAs are involved in regulating the expression of target genes the creation of mRNA precursors, such as miRNA and snRNA.

Supplementary Table 16. Functional annotation of genes from *G. japonicus*.

		Number of genes	Percentages in gene set
**Total**		22487	
	InterPro	17,106	76.07
	GO	14,123	62.81
**Annotated**	KEGG	15,788	70.21
	Swissprot	19,855	88.3
	TrEMBL	21,101	93.84
**Unannotated**		1,106	4.92

The majority of the genes could be functionally annotated based on combination of different databases. Only 4.92% of genes could not be annotated using current databases.

Supplementary Table 17. Gene families in *G. japonicus* and other species.

Species	Total genes	Unclustered genes	Families	Unique families	Ave. genes per family
G. japonicus	22,487	2,726	9,356	280	2.11
Al. sinensis	22,124	3,086	9,123	361	2.09
An. carolinensis	17,767	644	8,629	20	1.98
P. sinensis	20,571	504	8,700	31	2.31
C. mydas	19,273	2,485	8,574	213	1.96
X. tropicalis	18,429	281	8,121	47	2.23
C. familiaris	19,258	403	9,544	17	1.98
O. latipes	19,671	1,442	7,999	131	2.28
H. sapiens	21,375	1,011	9,565	100	2.13
M. gallopavo	14,098	374	7,968	7	1.72
G. gallus	16,700	2,076	8,139	37	1.8
O. anatinus	17,920	1,984	8,483	88	1.88
D. rerio	26,046	1,432	8,909	174	2.76
M. musculus	22,927	1,090	9,575	96	2.28

The 22,487 genes from *G. japonicus* clustered into 9,356 families, 2,726 genes remained unclustered.

Supplementary Table 18. Orthologous gene pairs in *G. japonicus* and other species.

Species vs Species	Orthologous gene	Percent (%)	Identity	
			Mean	Median
G.jap vs *A.car*	11,513	64.80	72.37	79.01
G.jap vs *G.gal*	9,974	59.72	68.45	81.18
G.jap vs *H.sap*	11,463	53.63	66.13	72.73
G.jap vs *X.tro*	10,362	56.23	61.84	67.88
G.jap vs *O.lat*	9,292	47.24	57.5	79.66

These data indicate that the closest relationship is the exist between *G. japonicus and A. carolinensis. G. jap, Gekko japonicus; A. car, Anolis carolinensis; G. gal, Gallus gallus; H. sap, Homo sapiens; X. tro, Xenopus tropicalis; O. lat, Oryzias latipes.*

Supplementary Table 19. Expansion of *beta-keratin* genes in *G. japonicus* genome.

Species	Beta-keratins family member	Beta-keratins family member with functional motif	Distribution on scaffolds	Structure of genes
G. japonicus	71	43	48 on scaffold426,<br>Others on 6 scaffolds else	68 Single exons,<br>46 on scaffold426
A. carolinensis	23	16	All on GL343369.1	Single exons
A. sinensis	2	2	On two scaffolds	Single exons

The analysis revealed that the *beta-keratin* family in *G. japonicus* contained far more mumbers than those in other species, and the majority of the family members (48 beta-keratin genes) were clustered in only one scaffold (scaffold426).

Supplementary Table 20. Physical and biochemical characteristics of beta-keratins from *G. japonicus*.

Gene ID	S-core box (sequence similarity≥70%, 35 protiens)	Cys percentage （Cys>10%，19 proteins）	Gly percentage (Gly>15%，36 proteins)	pI （pI>7，34 proteins)	Wt (Wt<15000，58 proteins)	Annotation
G. japonicus 19-D44	0.8	21.05	7.37	5.96	9184.79	beta-keratin 7 like
G. japonicus 18-D25	0.85	20.83	9.38	7.84	9447.28	beta-keratin 5 like
G. japonicus 19-D46	0.65	19.82	9.91	7.23	10710.76	beta-keratin 21 like
G. japonicus 18-D30	1	19.81	12.26	7.82	10394.28	beta-keratin 12 like
G. japonicus 18-D49	1	19.81	12.26	8.06	10390.32	beta-keratin 21
G. japonicus 18-D38	0.65	19.33	10.08	7.22	11774.77	beta-keratin 21 like
G. japonicus 16-D45	0.7	19.13	10.43	7.41	11332.18	beta-keratin 21 like
G. japonicus 16-D55	0.75	19.05	13.33	6.03	10080.67	beta-keratin 21 like
G. japonicus 19-D41	0.95	18.45	11.65	7.45	9958.71	beta-keratin 21
G. japonicus 18-D54	0.7	18.27	10.58	8.28	10332.4	beta-keratin 21 like
G. japonicus 18-D15	0.85	17.92	12.26	8.11	10330.19	beta-keratin 21
G. japonicus 15-D13	0.85	17.12	12.61	7.8	10766.67	beta-keratin 21
G. japonicus 18-D29	1	16.87	10.84	7.35	8171.6	beta-keratin 12
G. japonicus 15-D6	0.85	16.53	9.92	8.05	11831.94	beta-keratin 6
G. japonicus 15-D35	0.9	16.35	11.54	7.3	10079.78	beta-keratin 16
G. japonicus 15-D28	0.9	15.84	11.88	7.32	9820.46	beta-keratin 16
G. japonicus 17-D52	0.9	12.96	11.11	4.68	10587.19	beta-keratin 21 like
G. japonicus 2-D67	0.6	11.86	10.17	4.14	6224.17	beta-keratin 2 like
G. japonicus 15-D21	0.6	11.4	18.42	6.06	10862.45	beta-keratin 23
G. japonicus 15-D37	0.55	9.65	18.42	6.07	10809.36	beta-keratin 23
G. japonicus 5-D56	0.9	9.62	9.62	4.53	5138.03	beta-keratin 21
G. japonicus 13-D7	0.5	9.26	15.74	8.12	10418.89	beta-keratin 23 like
G. japonicus 6-D46	0.75	8.93	12.5	4.53	5434.27	beta-keratin 20 like
G. japonicus 4-D49	1	8.47	10.17	6.11	5810.73	beta-keratin 12
G. japonicus 10-D68	0.7	8.33	10	6.71	5937.88	beta-keratin 12 like
G. japonicus 4-D44	0.9	8.33	10	5.4	5852.72	beta-keratin 21
G. japonicus 4-D45	0.9	8.33	10	5.4	5852.72	beta-keratin 21
G. japonicus 5-D60	0.9	8.33	10	6.71	5897.72	beta-keratin 21
G. japonicus 6-D43	0.95	7.84	9.8	4.51	5045.87	beta-keratin 12
G. japonicus 5-D50	0.9	7.69	9.62	4.53	5121.97	beta-keratin 21
G. japonicus 5-D61	1	7.69	9.62	4.53	5121.97	beta-keratin 12
G. japonicus 8-D56	1	7.69	9.62	4.53	5137.97	beta-keratin 12
G. japonicus 3-D48	A Box	7.55	11.32	4	5096.08	beta-keratin 22 like
G. japonicus 1	0.7	7.45	17.02	8.59	9108.46	beta-keratin 22
G. japonicus 5-D58	0.7	6.67	10	6.72	5990.98	beta-keratin 13 like
G. japonicus 5-D62	0.9	6.67	10	6.12	5885.73	beta-keratin 13
G. japonicus 5-D63	0.9	6.67	10	6.12	5885.73	beta-keratin 13
G. japonicus 4-D15	0.55	6.45	16.13	7.74	9770.96	claw keratin-like
G. japonicus 5-D21	0.7	6.19	18.56	3.8	9063.42	beta-keratin 22 like
G. japonicus 1-D7	0.6	5.98	19.66	8.95	11730.42	beta-keratin 22 like
G. japonicus 1-D65	0.8	5.63	15.49	3.79	6772.83	beta-keratin 22 like
G. japonicus 20-D9	0.55	4.62	12.31	4.68	6601.49	claw keratin-like
G. japonicus 20-D10	0.6	3.8	25.95	8.22	15449.39	claw keratin-like
G. japonicus 8-D3	A Box	3.57	26.19	8.64	16004.25	beta-keratin 17 like
G. japonicus 1-D2	A Box	3.57	20.24	8.9	16102.23	beta-keratin 17 like
G. japonicus 8-D8	A Box	3.55	26.63	8.31	16240.42	beta-keratin 17 like
G. japonicus 17-D26	A Box	3.53	14.12	3.57	8066.2	beta-keratin 17 like
G. japonicus 5-D6	A Box	3.43	27.43	8.31	16640.8	beta-keratin 17 like
G. japonicus 6-D7	A Box	3.41	27.27	8.86	16906.21	beta-keratin 17 like
G. japonicus 9-D23	0.6	3.36	32.77	3.79	10984.21	beta-keratin 22 like
G. japonicus 6-D17	A Box	3.35	26.82	8.62	16829.16	beta-keratin 17 like
G. japonicus 1-D3	A Box	3.3	22.53	9.1	17338.52	beta-keratin 17
G. japonicus 3-D3	A Box	3.23	22.58	8.74	14683.73	beta-keratin 17 like
G. japonicus 1-D52	A Box	3.15	24.41	9.69	12185.78	beta-keratin 2 like
G. japonicus 22	0.55	3.08	19.23	4.36	12932.61	claw keratin-like
G. japonicus 20-D5	0.7	2.97	23.76	7.91	9839.92	beta-keratin 2 like
G. japonicus 2-D10	A Box	2.96	27.81	8.72	16158.34	beta-keratin 17 like
G. japonicus 3-D13	A Box	2.86	28	6.06	16488.71	beta-keratin 17 like
G. japonicus 20-D4	0.6	2.6	25.32	8.76	15203.05	beta-keratin 2 like
G. japonicus 1-D64	0.55	2.6	20.78	4.79	7838.59	scale keratin like
G. japonicus 6-D55	0.7	2.52	26.89	4.53	11267.45	beta-keratin 2 like
G. japonicus 6-D61	0.7	2.34	27.34	4.53	12142.4	beta-keratin 2 like
G. japonicus 9-D21	0.6	2.31	34.62	4.53	11973.31	beta-keratin 22 like
G. japonicus 1-D62	A Box	2.17	22.83	5.99	8606.8	beta-keratin 2 like
G. japonicus 1-D60	A Box	2.08	22.92	5.99	9227.54	beta-keratin 17 like
G. japonicus 1-D63	A Box	2.05	21.23	7	14053.96	beta-keratin 14 like
G. japonicus 5-D16	A Box	1.9	28.48	4.94	14622.44	beta-keratin 2 like
G. japonicus 3-D31	A Box	1.89	20.75	9.42	15242.27	beta-keratin 17 like
G. japonicus 3-D16	A Box	1.72	21.84	9.11	16372.49	beta-keratin 17
G. japonicus 8-D6	A Box	1.67	27.5	6.88	11156.53	beta-keratin 14 like
G. japonicus 8-D15	A Box	1.63	15.45	7.86	12120	beta-keratin 17 like

Many of the listed proteins include the S-core boxes, are rich in Cys and Gly, and possess higher isoelectric point (PI) and lower molecular weights (Wt).

Supplementary Table 21. Three gene families related to vision in *G. japonicus, A. carolinensis, G. gallus, H. sapiens* and *X. tropicalis.*

Gene families	*Gekko japonicus*	*Anolis carolinensis*	*Gallus gallus*	*Homo sapiens*	*Xenopus tropicalis*
**GPCR**	168	191	157	177	190
**INAD**	1	6	1	1	1
**PLC beta**	0	1	2	1	1

G. japonicus has less family members in all three gene families compared with its close relative *A. carolinensis*.

Supplementary Table 22. Opsins in *G. japonicus* and *A. carolinensis.*

Species	Opsin	Database ID	SwissProt ID	SwissProt Annotation
Anolis carolinensis		ENSACAP00000003346	Q9H1Y3	OPN3_HUMAN Opsin-3 OS=Homo sapiens GN=OPN3 PE=1 SV=1
		ENSACAP00000005696	Q7T3Q7	OPSO_RUTRU Opsin-VA OS=Rutilus rutilus PE=2 SV=1
		ENSACAP00000006735	P51476	OPSP_COLLI Pinopsin OS=Columba livia PE=1 SV=1
		ENSACAP00000007936	O14718	OPSX_HUMAN Visual pigment-like receptor peropsin OS=Homo sapiens GN=RRH PE=1 SV=1
		ENSACAP00000008376	P51476	OPSP_COLLI Pinopsin OS=Columba livia PE=1 SV=1
	**SWS1**	ENSACAP00000008716	P51491	OPSB_MOUSE Short-wave-sensitive opsin 1 OS=Mus musculus GN=Opn1sw PE=1 SV=1
		ENSACAP00000011614	O42266	OPSP_ICTPU Parapinopsin OS=Ictalurus punctatus PE=2 SV=1
	**SWS2**	ENSACAP00000012339	P28682	OPSB_CHICK Blue-sensitive opsin OS=Gallus gallus PE=1 SV=1
	**LWS**	ENSACAP00000012416	P41592	OPSR_ANOCA Red-sensitive opsin OS=Anolis carolinensis PE=1 SV=1
		ENSACAP00000013257	Q6U736	OPN5_HUMAN Opsin-5 OS=Homo sapiens GN=OPN5 PE=1 SV=3
		ENSACAP00000013346	Q4U4D2	OPN4_PODSI Melanopsin OS=Podarcis sicula GN=OPN4 PE=2 SV=2
	**RH1**	ENSACAP00000014023	P41591	OPSD_ANOCA Rhodopsin OS=Anolis carolinensis GN=RHO PE=2 SV=1
		ENSACAP00000014195	Q2KNE5	OPN4A_DANRE Melanopsin-A OS=Danio rerio GN=opn4a PE=2 SV=3
		ENSACAP00000015111	P47804	RGR_HUMAN RPE-retinal G protein-coupled receptor OS=Homo sapiens GN=RGR PE=1 SV=1
		ENSACAP00000015394	Q6VZZ7	OPN5_MOUSE Opsin-5 OS=Mus musculus GN=Opn5 PE=1 SV=1
	**RH2**	ENSACAP00000015846	P51471	OPSB_ANOCA Blue-sensitive opsin OS=Anolis carolinensis PE=1 SV=1
		ENSACAP00000017994	P51476	OPSP_COLLI Pinopsin OS=Columba livia PE=1 SV=1
		ENSACAP00000018012	O14718	OPSX_HUMAN Visual pigment-like receptor peropsin OS=Homo sapiens GN=RRH PE=1 SV=1
		ENSACAP00000019226	Q6VZZ7	OPN5_MOUSE Opsin-5 OS=Mus musculus GN=Opn5 PE=1 SV=1
		ENSACAP00000020520	Q6VZZ7	OPN5_MOUSE Opsin-5 OS=Mus musculus GN=Opn5 PE=1 SV=1
Gekko japonicus	**SWS1**	*GJA007273.1*	P51491	OPSB_MOUSE Short-wave-sensitive opsin 1 OS=Mus musculus GN=Opn1sw PE=1 SV=1
	**LWS**	*GJA008044.1*	P35358	OPSG_GECGE Green-sensitive opsin P521 OS=Gecko gecko PE=1 SV=1
		GJA009917.1	P51476	OPSP_COLLI Pinopsin OS=Columba livia PE=1 SV=1
	**RH2**	*GJA011086.1*	P35357	OPSB_GECGE Blue-sensitive opsin P467 OS=Gecko gecko PE=1 SV=1
		GJA012534.1	P47804	RGR_HUMAN RPE-retinal G protein-coupled receptor OS=Homo sapiens GN=RGR PE=1 SV=1
		GJA013390.1	Q6VZZ7	OPN5_MOUSE Opsin-5 OS=Mus musculus GN=Opn5 PE=1 SV=1
		GJA015083.1	Q7T3Q7	OPSO_RUTRU Opsin-VA OS=Rutilus rutilus PE=2 SV=1
		GJA016502.1	Q9H1Y3	OPN3_HUMAN Opsin-3 OS=Homo sapiens GN=OPN3 PE=1 SV=1
		GJA021990.1	Q4U4D2	OPN4_PODSI Melanopsin OS=Podarcis sicula GN=OPN4 PE=2 SV=2

The above data reveal that *A. carolinensis*, adiurnal reptile with tetrachromatic color vision has a greater quantity of light-sensitive opsins than *G. japonicus*, a nocturnal reptile.

Supplementary Table 23. Amino acid at site 89 in RH2, SWS1 and LWS/MWS of *G. japonicus*.

**Species**	RH2 F89C	SWS1 F89V	LWS I89V
G. Japonicus	---C---	---V---	---V---
A. carolinensis	---F---	---F---	---I---
C. mydas	---F---	---F---	---I---
T. guttata	---F---	---F---	---I---
G. gallus	---F---	---L---	---I---
O. latipes	---F---	---F---	---F---

Alignment of the amino acid sequences of the RH2, SWS1, and LWS/MWS pigments of *G. japonicus*, *A. carolinensis*, *C. mydas*, *T. guttata*, *G. gallus*, and *O. latipes* showed the varied amino acid at site 89.

Supplementary Table 24. Qualities of genome assemblies in seven species.

Species	Size of genome	Sequencing method and Sequencing depth	Sequencing depth	Contig assembly	Scaffold assembly	Positioning to chromosome
T. rubripes	380M	Sanger whole-genome shotgun	6X	Contig N50: 53Kb	Scaffold N50: 928Kb	Yes
D. rerio	1.4G	Clone sequence & WGS	45X	Contig N50: 1.26Mb	Scaffold N50: 1.55Mb	Yes
X. tropicalis	1.7G	Sanger whole-genome shotgun	7.6X	Contig N50: 17Kb	Scaffold N50: 1.6Mb	No
G. japonicus	2.55G	IlluminaHiseq 2000 WGS	131X	Contig N50: 21.1 Kb	Scaffold N50: 680 Kb	No
A. carolinensis	1.78G	Sanger whole-genome shotgun	6X	Contig N50:79.9Kb	Scaffold N50：4Mb	Yes
A. sinensis	2.3G	IlluminaHiseq 2000 WGS	136X	Contig N50:23.4kb	Scaffold N50：2.2 Mb	No
H. sapiens	2.9G	Clone sequence & WGS	38.7X	Contig N50: 23Kb	Scaffold N50: 29Mb	Yes

The summarized assembly quality parameters could be used to evaluate the risk of comparative analysis of olfactory receptor genes from these species.

Supplementary Table 25. Functional olfactory receptor genes in seven species.

Species	α	β	δ	ε	ζ	γ	Class I ORs	Class II ORs
T. rubripes	0	1	31	1	6	0	39	0
D. rerio	0	2	59	12	25	1	98	1
X. tropicalis	10	9	20	10	0	344	49	344
G. japonicus	40	2	0	0	0	251	42	251
A. carolinensis	1	0	0	0	0	87	1	87
A. sinensis	54	0	0	0	0	500	54	500
H. sapiens	57	0	0	0	0	404	57	404

The table listss all types of olfactory receptors from seven different species. Olfactory receptor genes significantly expand in *G. japonicus*, especially Class II OR genes, which mediate olfaction of molecules in the air. Approximately 3-fold more OR genes exist in *G. japonicus* (251 OR genes) than in *A. carolinensis* (87 OR genes).

Supplementary Table 26. A total of 155 PSGs were identified in *G. japonicus* based on background of six reptile species.

Gene ID	Annotation	Gene ID	Annotation
GJA001278.1	AASS	*GJA022967.3*	MYBBP1A
GJA019475.1	ADAM28	*GJA023575.2*	NAA40
GJA000565.1	AK7	*GJA018278.1*	NCAPG
GJA006867.1	ALG13	*GJA026182.1*	NIN
GJA016836.1	ALPK1	*GJA006002.2*	NOL10
GJA023434.1	ANXA1	*GJA001637.1*	NOP14
GJA016774.1	APOB	*GJA023685.1*	NRIP1
GJA009331.1	ARHGEF37	*GJA012178.1*	NUAK1
GJA017415.1	ASAH2	*GJA012626.1*	OBFC1
GJA003035.2	C10ORF71	*GJA004438.1*	P2RX3
GJA012845.3	C19orf21	*GJA024042.3*	PARP8
GJA025612.1	C5orf42	*GJA013398.1*	PC
GJA010414.3	CCDC114	*GJA006549.1*	PCSK1
GJA026897.1	CCDC164	*GJA006687.1*	PDHA1
GJA018805.1	CCDC173	*GJA006886.1*	PDIA3
GJA013250.1	CCDC39	*GJA016244.1*	PDYN
GJA000121.1	CCDC63	*GJA008938.3*	PEX5
GJA011277.1	CCDC66	*GJA018558.1*	PGM2L1
GJA008885.1	CCDC96	*GJA019219.1*	PLAU
GJA001605.1	CD3E	*GJA007008.2*	PLBD1
GJA004357.1	CDK7	*GJA027048.1*	PLG
GJA020456.1	CEP97	*GJA004397.1*	POLR1A
GJA007375.1	CHST4	*GJA007873.1*	PPP1R3A
GJA003670.1	CLPTM1	*GJA004149.2*	PPP5C
GJA002717.1	COL5A1	*GJA011671.1*	PTCD1
GJA004917.3	COL7A1	*GJA010344.1*	PTGIS
GJA003026.1	CRYBG3	*GJA002607.1*	PTGS1
GJA025998.1	CSPG4	*GJA008779.1*	PTPMT1
GJA001208.1	CTSC	*GJA001131.1*	QFLA-11381
GJA020099.1	CTSE	*GJA003399.1*	RAI1
GJA013001.2	DAG1	*GJA025325.1*	RECQL4
GJA013954.1	DCAF5	*GJA016007.1*	RET
GJA024629.1	DDX27	*GJA006752.4*	REV3L
GJA020293.1	DECR1	*GJA010900.1*	RHPN2
GJA023857.1	DIS3L	*GJA003121.1*	RMI1
GJA019286.1	DLEC1	*GJA011215.1*	RNF220
GJA003632.1	DNAJC9	*GJA006511.1*	RNF32
GJA008178.1	DUS3L	*GJA010672.3*	RPS6KA4
GJA010137.2	ECE2	*GJA015326.1*	RRNAD1
GJA024686.1	EIF2S2	*GJA006560.1*	SC5DL
GJA003759.3	EIF5B	*GJA020994.1*	SCARB2
GJA007967.1	EMILIN3	*GJA007108.1*	SCGN
GJA018053.1	ERP27	*GJA020989.1*	SDAD1
GJA022198.1	ESPL1	*GJA001258.1*	SERINC2
GJA025420.1	EXOC1	*GJA000090.1*	SKIDA1
GJA010491.1	F13A1	*GJA022460.1*	SLC18A3B
GJA025202.1	FAM124B	*GJA022797.1*	SLC1A1
GJA003166.1	FAM134A	*GJA019671.1*	SLC25A32
GJA011278.3	FAM208A	*GJA000859.1*	SLC26A8
GJA007025.1	FAM81B	*GJA008775.1*	SLC39A13
GJA022524.1	FAN1	*GJA003440.1*	SLC4A1
GJA023662.1	FBXO33	*GJA006565.2*	SPATA18
GJA014172.1	FGA	*GJA002540.1*	SRPR
GJA026816.1	FGD6	*GJA005980.1*	SSPN
GJA017780.1	FHAD1	*GJA009405.1*	SYNE1
GJA000097.1	FICD	*GJA007299.1*	TAF1
GJA022561.1	FNDC1	*GJA004396.1*	TBR1
GJA011799.1	GCNT3	*GJA015869.1*	TBRG4
GJA019054.1	GOLGB1	*GJA007288.1*	TEX11
GJA006088.2	GPR156	*GJA010123.1*	TGFBI
GJA002495.1	HCN4	*GJA014310.2*	TMBIM6
GJA014781.1	HOXA11	*GJA023189.1*	TMED7
GJA009117.1	IRAK1BP1	*GJA011217.1*	TMEM53
GJA007161.1	IRF3	*GJA015807.1*	TOMM22
GJA021603.2	IRX2	*GJA013113.1*	TTLL5
GJA022412.1	IYD	*GJA025511.3*	URB2
GJA004367.1	KCNA4	*GJA008389.1*	URI1
GJA004743.1	KIF15	*GJA018039.1*	UTP20
GJA008822.1	KIF20B	*GJA020984.1*	UTP3
GJA020665.1	KLB	*GJA016784.2*	UVSSA

GJA002015.1	KLF15	*GJA025846.1*	VCPIP1
GJA022254.1	KNTC1	*GJA017824.1*	VPS16
GJA015729.1	LACTB	*GJA008616.1*	VTN
GJA000593.1	MFI2	*GJA008897.2*	WDR62
GJA008410.1	MFRP	*GJA023512.1*	XDH
GJA008832.1	MINPP1	*GJA025436.1*	ZC3H15
GJA001164.1	MPST	*GJA014527.1*	ZNF518A
GJA007726.1	MRPL32		

Differences in the mean Ka/Ks ratio for single-copy genes between *Gekko japonicus* and each of the other species were compared using paired Wilcoxon rank sum tests. After filtering out the false positive genes, we obtained a final total of 155 positive selection genes in *Gekko japonicus*.

Supplementary Methods

Genome sequencing, size evaluation and quality estimation

Genomic DNA was extracted from the blood of an adult male *Gekko japonicus* sampled in Jiangsu, China. The DNA was fragmented and different insert sizes by were isolated electrophoresis. PCR amplification was performed following the addition of adapters, and the products were clustered to form mate-pair libraries (insert-size ≥ 2Kb). A total of 12 libraries including for sizes of 170bp, 500bp, 800bp, 2Kb, 5Kb, 10Kb, 20Kb and 40Kb were constructed. This facilitated a high level of genome assembly to create a N50 length scaffold. The genomic DNA was sequenced using an Illumina HiSeq™ 2000 platform (20 lanes, 330.90 Gb, 131.35X). Reads were discarded when they contained one of the following: 1) greater than 2% Ns or with poly(A) structure; 2) greater than 40% low quality bases (quality value < 8) for small insert-size libraries and 60% for large insert size libraries; 3) adapter pollution; 4) overlaps between read 1 and subsequent reads (at least 10 bp overlap, with < 10% mismatch); 5) duplicated reads within fastq files. The clean data comprised 233.49 Gbp, which covered the genome by 94.65-fold, and the physical coverage was approximately 1693.27-fold. Approximately 0.14% of the small-insert-size clean data were further corrected according to high frequency and low frequency K-mers defined by K-mer distributions (frequency ≤ 11 was considered low-frequency for the17-mers), and 0.47% of the reads and 1.95% of the bases were deleted. Genome size, heterozygosis and repeat content were estimated using K-mer analysis. Reads comprising 87.14 Gbp of clean data (34.52X) were split into K-mers, and K-mer depth was computed to obtain depth distribution[1]. The peak K-mer depth is about 29, which was determined to be the K-mer expectation depth. Genome size was estimated as K-mer num/Peak depth, which results 2.52 Gb with 660033 scaffolds.

SOAPdenovo (v2.04)[2] and *SSPACE*[3] software were used for genome to assembly. The clean data corresponding to the short-insert-size library were split into 29-mers, used to construct a de Bruijn graph, and connected to obtain contigs. Scaffolds were constructed by realigning all usable reads onto the contig sequences, calculating the amount of shared paired-end relationships between each pair of contigs, and linking the contigs into scaffolds using *SOAPdenovo* followed with *SSPACE*. The read pairs were finally retrieved, and one was mapped to the unique contig. Local assembly was performed to fill the gaps with the other read by pair end relation. The contig N50 and scaffold N50 following the final assembly were 21.07 Kb and 684.96 Kb respectively**.** All reads were aligned to the assembled scaffolds using *SOAPaligner2*[4], and the depth

of each base was calculated using soap.coverage program with an average assembly depth of approximately 80-89X. After graphing the GC depth, the GC content was found to be approximately 45%.

To assess the accuracy of the assembly, 10 fosmids were sequenced and aligned to the assembled scaffolds[5]. Plasmid DNA was extracted and sheared into fragments approximately 1~3Kb in length using Gene Machines. The DNA fragments were purified and end-repaired to blunt, followed by ligation into the PUC118-Vector and transformation into *Escherichia coli* by electroporation. The transformants were plated on X-gal and IPTG LB plates with ampicillin overnight. Large-scale Sanger sequencing was performed on an ABI 3730. When the sequence coverage reached to about 6 folds, the assembly process was initiated, and more Sanger reads were sequenced to get the complete map. The 10 fosmid clones were therefore used as reference data, and they were used to map the assembled genome sequence (BLASTn, E-value threshold of 1e-5) to check the coverage rate. The alignments were contiguous and of high quality, and the average coverage rate of the fosmid clones was about 99.65%.

The completeness of the euchromatic portion of the assembly was assessed using RNA-Seq data. The EST of *Gekko japonicus* was downloaded from NCBI. The Illumina RNA-Seq reads data were assembled into unigenes by Trinity[3], and TGICL[6] was used to remove redundancy. The EST and the unigenes built by Trinity were aligned on the assembled scaffolds using BLAT with an identity cutoff of 90%.

Transcriptome sequencing

Transcriptome analysis included two experiments. The first was a multiple tissues assay to produce a *G. japonicus* gene set. Brain, spinal cord, liver, and ovary were pooled for RNA sequencing. The second was the creation of an expression profile of the tail at different time point after amputation. For sampling, 50 individuals were randomly divided into 5 groups with half males and half females in each group by the random number table method. A caudectomy was conducted by inserting a nylon slipknot at the site of the sixth tail segment and pulling gently, which mimicked the conditions of natural autotomy. The endmost tissues of the tail were sampled at a length of 0.5 cm on days 0, 1, 3, 7 and 14, and 10 individuals (half males and half females) were prepared for each time point. The samples were then submitted to RNA sequencing. RNA sequencing was conducted as follows. Oligo(dT) beads were used to enrich poly(A) mRNA after total RNA was collected from the above samples.

Fragmentation buffer was added to cut the mRNA into short fragments, which were used as templates. Random hexamer primers were used to synthesize first-strand cDNA. Second-strand cDNA was synthesized using a mixture of buffer, dNTPs, RNase H and DNA polymerase I. Short fragments were purified with QiaQuick PCR extraction kits and resolved with EB buffer for end repair and addition of poly(A). Next, the short fragments were connected using sequencing adapters. For amplification with PCR, suitable fragments were selected as templates based on agarose gel electrophoresis. Finally, the libraries were sequenced on a Illumina HiSeq™ 2000.

Dirty reads were discarded from the raw data when one of the following conditions were met: 1) contain adapter sequences, 2) ambiguous nucleotides comprised more than 5%, 3) low quality reads comprised more than 30% and 4) quality less than 10. Clean reads were mapped to the reference genomes and gene sequences using *SOAP*(v.2.21). Mismatches of no more than 5 bases were allowed in the alignment. The proportion of clean reads mapped back to the genome and genes, provided an overall assessment of sequencing quality.

Segment duplication

LASTZ (T: 2, C:2, H:2000, Y:3400, L:6000, K:2200) was used to identify the segment duplication blocks in *Gekko japonicus*. There were 39,863 duplicate blocks longer than 1Kb in the genome, covering about 53 Mb of the genome in total. The longest block is 217,810 bp. The majority of the blocks are between 1 Kb and 5 Kb in length. The identity is greater than 98%.

Repeat annotation

Repeats were identified by a combination of homology-based and de novo prediction approaches. The homology-based approach found out known repeats using Repbase[7] by RepeatMasker[8] and RepeatProteinMask (http://www.repeatmasker.org). *De novo* prediction was performed using RepeatMakser on repeat libraries generated by *RepeatModeler* (http://repeatmasker.org). Additionally, tandem repeats were identified using program *Tandem Repeats Finder* (v.4.04)[9].

Gene prediction and functional annotation

Gene prediction was performed using GLEAN(http://sourceforge.net/projects/glean-

gene) integration of *de novo* and homologous gene models, and RNA-seq data were applied to refine the gene set. *De novo* prediction was performed based on the repeat-masked genome. Two programs *AUGUSTUS* (v.2.5.5)[10] and *GENSCAN*[11] were used for the prediction. Homolog-based prediction was performed by mapping protein sequences (downloaded from NCBI) of related representative species (*Anolis carolinensis, Gallus gallus, Homo sapiens, Meleagris gallopavo* and *Xenopus tropicalis*) to the genome using *TblastN* (E-value cutoff 1e-5). Alignment and identification of accurate spliced alignments was accomplished using *GeneWise* (wise2-2-0)[12]. The EST of *Gekko japonicus* (download from NCBI) was aligned against the assembly genome using BLAT (identity >= 0.95, coverage >= 0.90) to generate spliced alignments, and PASA was used to filter the overlaps, link the spliced alignments and predict the possible gene model. The data were integrated by *GLEAN* to produce a consensus gene set. Also, transcriptomes data from multiple tissues were aligned to genome using tophat and assembled using cufflinks to improve the accuracy and completeness of the predicted gene set[13,14]. Finally, a total of 22,487 genes were obtained, with an average exons number per gene of 7.90, an average gene length of 25,676 bp, and an average CDS length of 2,426 bp. These values were similar between the closely related species. Gene functions were annotated based on the best matched hits to SwissProt and TrEMBL databases (Release15.10) using BLASTp (E-value<=1e-5). Gene motifs and domains were identified by InterProScan (v.4.7) against protein databases. All genes were aligned against KEGG databases proteins (E-value<=1e-5), and the pathwaysthat the genes might be involved in were derived based on the matching genes in the KEGG database.

Phylogenetic analysis

A phylogenetic tree was constructed using single-copy orthologous genes from *Alligator sinensis, Anolis carolinensis, Pelodiscus sinensis, Chelonia mydas, Xenopus tropicalis, Canis familiaris, Oryzias latipes, Homo sapiens, Meleagris gallopavo, Gallus gallus, Ornithorhynchus anatinus, Danio rerio, Danio rerio, Taeniowgia guttata, Python molurus bivittatus*and *Gekko japonicus*. Each gene from an orthologous genes family was subjected to multiple sequence alignment using MUSCLE[15], and concatenated into a super sequence. PhyML was used to construct the phylogenetic tree under the GTR and invgamma model functions[16]. The same sequence set was applied to estimate the periods of species divergence using the program PAML *MCMCTREE* under the correlated molecular clock function in the approximate likelihood calculation method[17]. The correlated molecular clock and REV substitution model were selected to perform estimation. The MCMC process of PAML

mcmctree was run to sample 100,000 times with a sample frequency of 50 after a burn-in of 5,000,000 iterations. Fossil calibrates were derived from www.fossilrecord.net. For calibration, divergence periods with paleontological evidence were used to date the nodes of the tree between two species[18] (*Taeniopygia guttata-Alligator sinensis* 250.4-235 Mya, *Gallus gallus-Anolis carolinensis* 299.8-259.7 Mya, *Homo sapiens-Mus musculus* 100.5-61.5 Mya, *Homo sapiens-Ornithorhynchus anatinus* 191.1-162.5 Mya, *Pelodiscus sinensis-Homo sapiens* 330.4-312.3 Mya ,*Gallus gallus- Taeniopygia guttata* 86.5-66 Mya, *Oryzias latipes-Danio rerio* 165.2-149.85 Mya).

Gene family identification

Gene families were identified using *TreeFam*[19]. All-versus-all BLASTP alignment of protein sequences of 14 species (*Al. sinensis*, *An. carolinensis*; *P. sinensis*, *C. mydas*, *X. tropicalis*, *C. familiaris*, *O. latipes*, *H. sapiens*, *M. gallopavo*, *G. gallus*, *O. anatinus*, *D. rerio*, *M. musculus*, and *G. japonicus*) was performed with an E-value less than 1e-7. Then, HSP segments were concatenated between the same pair of proteins with Solar, and similarities were evaluated by Bit-score. Gene families were determined by *hcluster_sg* (v.0.5.0) clustering[1]**.** The 22,487 genes clustered to 9,356 families and 2,726 genes remained unclustered.

Expansion and contraction analysis of gene families

Gene family expansion and contraction were identified using CAFÉ[20] , which employed a random birth and death model to study gene gain and loss in gene families across a user-specified phylogeny. The global parameter λ, which described both the gene birth (λ) and death (μ = -λ) rate across all branches in the tree for all gene families, was estimated using maximum likelihood. A conditional p-value was calculated for each gene family, and families with conditional p-values under the threshold (0.0001) were considered to have accelerated rates of gain or loss. We identified branches responsible for low overall p-values of significant families. A total of 2694 contracted families involving 4602 genes were identified, and 1247 expanded families with 5574 genes were also identified.

Positive selection genes

Ka/ks ratios were calculated for all single copy orthologs of *Gekko japonicus*, *Anolis carolinensis*, *Alligator sinensis, Python molurus bivittatus, Chelonia mydas,* and

Pelodiscus sinensis. Orthologous genes were first aligned by PRANK (v.100802)[21], an alignment tool used for studies of molecular evolution[22]. Gblocks (v.0.91b)[23,24] was used to remove ambiguously aligned blocks within PRANK alignments. The 'codeml' in the PAML package[17] with the free-ratio model was employed to estimate Ka, Ks, and Ka/Ks ratios on different branches. The difference in mean Ka/Ks ratio for single-copy genes between *Gekko japonicus* and each of the other species were compared with paired Wilcoxon rank sum tests[25]. After filtering out the false positive genes, we obtained a final total of 155 positive selection genes in *Gekko japonicus*.

Analysis of beta-keratins in setae

Beta-keratin proteins encoded in the *Anolis carolinensis* genome were used as a reference[26], and aligned with the genome sequence of *Gekko japonicus* and *Alligator sinensis* by tBLASTn (1e-5). Then, the genome sequence was analyzed with Genewise to predict candidate gene models, which were syntenic to the matched proteins, and gained the homolog genes. A conserved region among the 71 beta-keratin proteins of *Gekko japonicus* (TCISQCPPSSVFIQPPPFCVTVPGPIMSCADEPCAVECTTPCAP SY) was identified from the Muscle alignment and used for predictions with PHD software (http://npsa-pbil.ibcp.fr/).

Select amino acid sequences are characteristic of beta-keratins [27] including S-core box (SEVTIQPPPCTVVVPGPVLA), previously termed the Ge-cprp-9 core box, and the A Box (AEVLIQPPPSVVTLPGPILS), previously termed the Ge-gprp-6 core box. Both of these motifs exist in the digital pads of *G. gecko*[28]. S-core box containing proteins in particular have an extremely important role in setae present on digital pads of *Gekko gecko*[27].

The expansion time of beta-keratins in setae was evaluated as follows. Reptilian and avian beta-keratins share a common ancestor[29]. We constructed a phylogenetic tree of beta-keratin proteins from different species and calculated the divergence of each branch. The molecular clock of protein evolution is roughly constant according to neutral theory[30,31], therefore, a divergence value is proportional to the timescale of the corresponding genetic event. As such, we could calculate the timescales of unknown genetic events based on the dates of another known events. However, the actual evolution rate of a protein might fluctuate for various known and unknown reasons. Therefore, this method will not produce a reliable result unless appropriately calibrated time points are available. The timescale of avian scale and claw keratins divergence[32] and the timescale of feather keratins expansion in *Neornithes*[33] were used to calibrate

the expansion time of beta-keratins in setae. The average value of the two calibrating point were used for final evaluation. The resultant expansion time (105-96 Mya) perfectly corresponded to the emergence of the earliest known adhesive toe pads (110-97 Mya)[34] as dated by fossil evidence.

Analysis of light-sensitive opsins

An opsin domain published on Interpro with the ID IPR001760 was used as a reference (http://www.ebi.ac.uk/interpro/entry/ IPR001760). The opsin subtypes of *G. japonicus* and *A. carolinensis* were identified based on the motif and then checked in the SwissProt database and submitted to functional annotation. Sequences corresponding to GPCR[35], INAD[36] and PLCβ[37], which are visual proteins in *H. sapiens*, were downloaded from NCBI and further aligned with the gene set of a given species using BLASTp (1e-5; alignment coverage of the query and subject sequence greater than 70%).

To create an *opsins* phylogenetic tree, opsins from the following species were obtained from the UniProt database: *Drosophila melanogaster, Danio rerio, Oryzias latipes, Xenopus tropicalis, Chelonia mydas, Gallus gallus, Taeniopygia guttata, Anolis carolinensis, Gekko gecko,* and *Homo sapiens*, among other. Multiple sequence alignment was conducted using these protein sequences with MUSCLE[15]. A maximum likelihood estimate for the aligned sequences was obtained using PhyML under the WAG model.

Analysis of olfactory receptor (OR) genes

OR protein sequences from *H. sapiens* were downloaded from NCBI, and aligned with the gene sets from *Al. sinensis*, *An. carolinensis*, *P. sinensis*, *C. mydas*, *X. tropicalis*, *G. gallus* and *G. japonicus* by BLAST (1e-5; alignment coverage greater than 70%). Genes were identified as ORs if they possessed the OR motif (INTERPROSCAN). An OR family tree was constructed using OR proteins (a total of 1939 proteins) from*X. tropicalis*, *Al. sinensis*, *P. sinensis*, *C. mydas*, *An. carolinensis* and *G. japonicus* via the Neighbour-Joining method.

Gene Ontology (GO) functional enrichment analyses

GO functional enrichment analysis was used to map all genes to terms in the GO database (http://www.geneontology.org/) by calculating gene numbers for every term.

An ultra-geometric test was then used to identify significantly enriched GO terms in the target gene list compared to the genomic background. To accomplish this, the following formula was used:

$$P = 1 - \sum_{i=0}^{m-1} \frac{\binom{M}{i}\binom{N-M}{n-i}}{\binom{N}{n}}$$

where N represents the total number of genes with GO annotation, n represents the number of target genes in N, M represents the total number of genes that are annotated to certain GO terms, and m represents the number of target genes in M. The calculated p-value was submitted to Bonferroni correction with a threshold-corrected p-value ≤ 0.05. GO terms fulfilling this condition were considered to be significantly enriched[38].

Gene expression

Gene coverage was defined as the percentage of a gene covered by reads. This value was equivalent to the ratio of the number of bases in a gene covered by unique mapping reads to the number of total bases in that gene. Gene expression was calculated using the RPKM method (Reads Per Kb per Million reads)[39] as follows:

$$RPKM = \frac{10^6 C}{NL / 10^3}$$

where C represents the number of reads that uniquely aligned to gene A, N represents the total number of reads that uniquely aligned to all genes, and L represents the base number in the CDS of gene A. Using the RPKM method eliminated the influence of different gene lengths and sequencing discrepancies on gene expression calculations. Therefore, the above calculations were used to directly compare differences in gene expression among samples.

Supplementary References

1 Li, R. *et al.* The sequence and de novo assembly of the giant panda genome. *Nature* **463**, 311-317, doi:10.1038/nature08696 (2010).

2 Li, R. *et al.* De novo assembly of human genomes with massively parallel short read sequencing. *Genome Res* **20**, 265-272, doi:10.1101/gr.097261.109 (2010).

3 Boetzer, M., Henkel, C. V., Jansen, H. J., Butler, D. & Pirovano, W. Scaffolding pre-assembled contigs using SSPACE. *Bioinformatics* **27**, 578-579, doi:10.1093/bioinformatics/btq683 (2011).

4 Li, R. *et al.* SOAP2: an improved ultrafast tool for short read alignment. *Bioinformatics* **25**, 1966-1967, doi:10.1093/bioinformatics/btp336 (2009).

5 Zhang, L. *et al.* Fosmid library construction and initial analysis of end sequences in Zhikong scallop (Chlamys farreri). *Mar Biotechnol (NY)* **9**, 606-612, doi:10.1007/s10126-007-9014-4 (2007).

6 Pertea, G. *et al.* TIGR Gene Indices clustering tools (TGICL): a software system for fast clustering of large EST datasets. *Bioinformatics* **19**, 651-652 (2003).

7 Jurka, J. *et al.* Repbase Update, a database of eukaryotic repetitive elements. *Cytogenet Genome Res* **110**, 462-467, doi:10.1159/000084979 (2005).

8 Tarailo-Graovac, M. & Chen, N. Using RepeatMasker to identify repetitive elements in genomic sequences. *Curr Protoc Bioinformatics* **Chapter 4**, Unit 4 10, doi:10.1002/0471250953.bi0410s25 (2009).

9 Benson, G. Tandem repeats finder: a program to analyze DNA sequences. *Nucleic acids research* **27**, 573-580 (1999).

10 Stanke, M. & Morgenstern, B. AUGUSTUS: a web server for gene prediction in eukaryotes that allows user-defined constraints. *Nucleic acids research* **33**, W465-467, doi:10.1093/nar/gki458 (2005).

11 Burge, C. B. & Karlin, S. Finding the genes in genomic DNA. *Curr Opin Struct Biol* **8**, 346-354 (1998).

12 Birney, E., Clamp, M. & Durbin, R. GeneWise and Genomewise. *Genome Res* **14**, 988-995, doi:10.1101/gr.1865504 (2004).

13 Trapnell, C., Pachter, L. & Salzberg, S. L. TopHat: discovering splice junctions with RNA-Seq. *Bioinformatics* **25**, 1105-1111, doi:10.1093/bioinformatics/btp120 (2009).

14 Trapnell, C. *et al.* Differential gene and transcript expression analysis of RNA-seq experiments with TopHat and Cufflinks. *Nat Protoc* **7**, 562-578, doi:10.1038/nprot.2012.016 (2012).

15 Edgar, R. C. MUSCLE: multiple sequence alignment with high accuracy and high throughput. *Nucleic acids research* **32**, 1792-1797, doi:10.1093/nar/gkh340 (2004).

16 Guindon, S. *et al.* New algorithms and methods to estimate maximum-likelihood phylogenies: assessing the performance of PhyML 3.0. *Syst Biol* **59**, 307-321, doi:10.1093/sysbio/syq010 (2010).

17 Yang, Z. PAML: a program package for phylogenetic analysis by maximum likelihood. *Comput Appl Biosci* **13**, 555-556 (1997).

18 Benton, M. J. & Donoghue, P. C. Paleontological evidence to date the tree of life. *Molecular biology and evolution* **24**, 26-53, doi:10.1093/molbev/msl150 (2007).

19 Li, H. *et al.* TreeFam: a curated database of phylogenetic trees of animal gene families. *Nucleic acids research* **34**, D572-580, doi:10.1093/nar/gkj118 (2006).

20 De Bie, T., Cristianini, N., Demuth, J. P. & Hahn, M. W. CAFE: a computational tool for the study of gene family evolution. *Bioinformatics* **22**, 1269-1271, doi:10.1093/bioinformatics/btl097 (2006).

21 Loytynoja, A. & Goldman, N. Phylogeny-aware gap placement prevents errors in sequence alignment and evolutionary analysis. *Science* **320**, 1632-1635, doi:10.1126/science.1158395 (2008).

22 Markova-Raina, P. & Petrov, D. High sensitivity to aligner and high rate of false positives in the estimates of positive selection in the 12 Drosophila genomes. *Genome Res* **21**, 863-874, doi:10.1101/gr.115949.110 (2011).

23 Castresana, J. Selection of conserved blocks from multiple alignments for their use in phylogenetic analysis. *Molecular biology and evolution* **17**, 540-552 (2000).

24 Talavera, G. & Castresana, J. Improvement of phylogenies after removing divergent and ambiguously aligned blocks from protein sequence alignments. *Syst Biol* **56**, 564-577, doi:10.1080/10635150701472164 (2007).

25 Preuss, T. M. Human brain evolution: from gene discovery to phenotype discovery. *Proc Natl Acad Sci U S A* **109 Suppl 1**, 10709-10716, doi:10.1073/pnas.1201894109 (2012).

26 Alfoldi, J. *et al.* The genome of the green anole lizard and a comparative analysis with birds and mammals. *Nature* **477**, 587-591, doi:10.1038/nature10390 (2011).

27 Alibardi, L. Immunolocalization of specific keratin associated beta-proteins (beta-keratins) in the adhesive setae of Gekko gecko. *Tissue Cell* **45**, 231-240, doi:10.1016/j.tice.2013.01.002 (2013).

28 Hallahan, D. L. *et al.* Analysis of gene expression in gecko digital adhesive pads indicates significant production of cysteine- and glycine-rich beta-keratins. *J Exp Zool B Mol Dev Evol* **312**, 58-73, doi:10.1002/jez.b.21242 (2009).

29 Ye, C., Wu, X., Yan, P. & Amato, G. beta-Keratins in crocodiles reveal amino acid homology with avian keratins. *Mol Biol Rep* **37**, 1169-1174, doi:10.1007/s11033-009-9480-z (2010).

30 M., K. Molecular evolutionary clock and the neutral theory. *J Mol Evol* **J Mol Evol. 1987;26(1-2):24-33.**, J Mol Evol. 1987;1926(1981-1982):1924-1933. (1987).

31 FJ., A. The theory of evolution: the case for randomness in the evolution of DNA and proteins. "The neutral theory of molecular evolution." By Motoo Kimura. *Hist Philos Life Sci* **Hist Philos Life Sci. 1986;8(1):129-38.** (1986).

32 Greenwold, M. J. & Sawyer, R. H. Linking the molecular evolution of avian beta (beta) keratins to the evolution of feathers. *J Exp Zool B Mol Dev Evol* **316**, 609-616, doi:10.1002/jez.b.21436 (2011).

33 Zhang, G. *et al.* Comparative genomics reveals insights into avian genome evolution and adaptation. *Science* **346**, 1311-1320, doi:10.1126/science.1251385 (2014).

34 P., E. N. A. G. A 100 million year old gecko with sophisticated adhesive toe pads, preserved in amber from Myanmar. *Zootaxa* **1847: 62-68** (2008).

35 Augustin, R., O'Sullivan, S. & Davies, I. a. I. Isolation of grass pollen antigens failing to induce IgE reagin formation although capable of inducing IgG antibody formation. *Int Arch Allergy Appl Immunol* **41**, 144-147 (1971).

36 Montell, C. Dynamic regulation of the INAD signaling scaffold becomes crystal clear. *Cell* **131**, 19-21, doi:10.1016/j.cell.2007.09.022 (2007).

37 Lyon, A. M. & Tesmer, J. J. Structural insights into phospholipase C-beta function. *Mol*

Pharmacol **84**, 488-500, doi:10.1124/mol.113.087403 (2013).

38 Chen, L. *et al.* Gene Ontology and KEGG Pathway Enrichment Analysis of a Drug Target-Based Classification System. *PLoS One* **10**, e0126492, doi:10.1371/journal.pone.0126492 (2015).

39 Mortazavi, A., Williams, B. A., McCue, K., Schaeffer, L. & Wold, B. Mapping and quantifying mammalian transcriptomes by RNA-Seq. *Nat Methods* **5**, 621-628, doi:10.1038/nmeth.1226 (2008).
